# Loss of function of GATA3 regulates FRA1 and c-FOS to activate EMT and promote mammary tumorigenesis and metastasis

**DOI:** 10.1038/s41419-023-05888-9

**Published:** 2023-06-23

**Authors:** Xiong Liu, Feng Bai, Yuchan Wang, Chuying Wang, Ho Lam Chan, Chenglong Zheng, Jian Fang, Wei-Guo Zhu, Xin-Hai Pei

**Affiliations:** 1 Guangdong Provincial Key Laboratory of Regional Immunity and Diseases, International Cancer Center, Marshall Laboratory of Biomedical Engineering, The First Affiliated Hospital, Shenzhen University Health Science Center, Shenzhen, 518060 China; 2 Department of Pathology, Shenzhen University Health Science Center, Shenzhen, 518060 China; 3 Dewitt Daughtry Family Department of Surgery, University of Miami, Miami, FL 33136 USA; 4 Gansu Dian Medical Laboratory, Lanzhou, 730000 China; 5 The Second Affiliated Hospital of Xi’an Jiaotong University, Xi’an, Shaanxi 710061 China; 6 Department of Biochemistry and Molecular Biology, International Cancer Center, Shenzhen University Health Science Center, Shenzhen, 518060 China; 7 Department of Anatomy and Histology, Shenzhen University Health Science Center, Shenzhen, 518060 China

**Keywords:** Breast cancer, Cancer stem cells, Cancer stem cells, Oncogenesis

## Abstract

Basal-like breast cancers (BLBCs) are among the most aggressive cancers, partly due to their enrichment of cancer stem cells (CSCs). Breast CSCs can be generated from luminal-type cancer cells via epithelial-mesenchymal transition (EMT). GATA3 maintains luminal cell fate, and its expression is lost or reduced in BLBCs. However, deletion of Gata3 in mice or cells results in early lethality or proliferative defects. It is unknown how loss-of-function of GATA3 regulates EMT and CSCs in breast cancer. We report here that haploid loss of Gata3 in mice lacking p18Ink4c, a cell cycle inhibitor, up-regulates Fra1, an AP-1 family protein that promotes mesenchymal traits, and downregulates c-Fos, another AP-1 family protein that maintains epithelial fate, leading to activation of EMT and promotion of mammary tumor initiation and metastasis. Depletion of Gata3 in luminal tumor cells similarly regulates Fra1 and c-Fos in activation of EMT. GATA3 binds to FOSL1 (encoding FRA1) and FOS (encoding c-FOS) loci to repress FOSL1 and activate FOS transcription. Deletion of Fra1 or reconstitution of Gata3, but not reconstitution of c-Fos, in Gata3 deficient tumor cells inhibits EMT, preventing tumorigenesis and/or metastasis. In human breast cancers, GATA3 expression is negatively correlated with FRA1 and positively correlated with c-FOS. Low GATA3 and FOS, but high FOSL1, are characteristics of BLBCs. Together, these data provide the first genetic evidence indicating that loss of function of GATA3 in mammary tumor cells activates FOSL1 to promote mesenchymal traits and CSC function, while concurrently repressing FOS to lose epithelial features. We demonstrate that FRA1 is required for the activation of EMT in GATA3 deficient tumorigenesis and metastasis.

## Introduction

There is increasing evidence that cancer stem cells (CSCs) initiate tumors and are particularly radio- and chemo-resistant, driving metastasis and poor prognoses [1, 2]. CSCs are a subpopulation of cancer cells that share the properties of self-renewal and multipotency with stem cells. CSCs in carcinomas can be generated from non-stem cancer cells by epithelial-mesenchymal transition (EMT) [3–6]. EMT describes a process in which epithelial cells lose many of their epithelial characteristics and acquire mesenchymal features [6]. Breast cancer is mainly divided into estrogen receptor (ER) positive luminal and ER-negative basal-like tumors [7]. Basal-like breast cancers (BLBCs) are poorly differentiated and are the most lethal, partly due to their enrichment of CSCs [8–10]. We and others have demonstrated that at least some of the BLBCs originate from luminal epithelial cells or luminal tumor cells [11–16]. The molecular mechanisms controlling EMT and CSCs in breast cancers remain to be clarified.

Transcription factor GATA3 is essential in maintaining mammary luminal cell fate and promoting luminal cell differentiation [11–14, 17, 18]. High GATA3 expression is a feature of luminal-type breast cancer and predicts better survival [19, 20]. GATA3 is often silenced by DNA methylation [21, 22] and its expression is lost or significantly reduced in BLBCs [20, 23–25] and metastasized breast cancers [23]. Overexpression of GATA3 suppresses EMT in cancer cell lines [26, 27] and loss of Gata3 in oncogene transgenic mice stimulates mammary luminal tumor progression with expansion of stem cell-like tumor cells [28, 29]. However, germline or mammary epithelium-specific deletion of Gata3 [17, 18, 30, 31] in mice results in early lethality or growth defects and targeted deletion of GATA3 in tumor cells leads to apoptosis [28], making it difficult to determine the mechanism of loss-of-function of Gata3 in activation of EMT in mammary tumor initiation and progression. How loss-of-function of GATA3 regulates EMT and mammary CSCs in tumor initiation and progression remain elusive.

Activator protein-1 (AP-1) is a transcription factor that is a heterodimeric protein composed of proteins belonging to the FOS and JUN families. AP-1 complex plays a key role in activating EMT and driving breast CSC function [32–34]. c-FOS (encoded by FOS) is mainly expressed in mammary epithelial cells, activates E-cad transcription, and plays a critical role in maintaining the state of the epithelial cells. Whereas FRA1 (encoded by FOSL1), a member of the FOS family of transcription factors, preferentially expressed in CSCs and the mammary epithelial cells that have undergone EMT. FRA1 binds to JUN to form heterodimeric AP-1 complexes, maintaining mesenchymal cell state, inducing EMT and generating CSCs from non-stem cancer cells [33, 35]. During execution of the EMT program, c-FOS is down-regulated and FRA1 is up-regulated, and there is a switch from the use of c-FOS to FRA1 as the preferred component of AP-1 transcription factor complexes [35]. Notably, we previously demonstrated that the expression of FRA1 is elevated and EMT is activated in Gata3 deficient breast cancers [15]. How GATA3 regulates AP-1 complex in mammary tumorigenesis and metastasis remains elusive.

We previously demonstrated that p18^INK4C^ (p18), a cell cycle inhibitor, is a downstream target of GATA3 and restrains mammary epithelial cell (MEC) proliferation and tumorigenesis [20]. We discovered that depletion of Gata3 in p18 deficient mice results in BLBCs with EMT features and enrichment of CSC characteristics [15, 16]. Furthermore, depletion of Gata3 in luminal tumor cells activates EMT. By using these mouse models, we investigated how GATA3 regulates the AP-1 transcription factor and whether AP-1 is essential for GATA3 deficiency-activated EMT in mammary tumor development and progression.

## Results

### Haploid loss of Gata3 enhances Fra1 and reduces c-Fos expression leading to the activation of EMT and driving mammary tumor initiating and metastatic potential

**Fig. 1:**
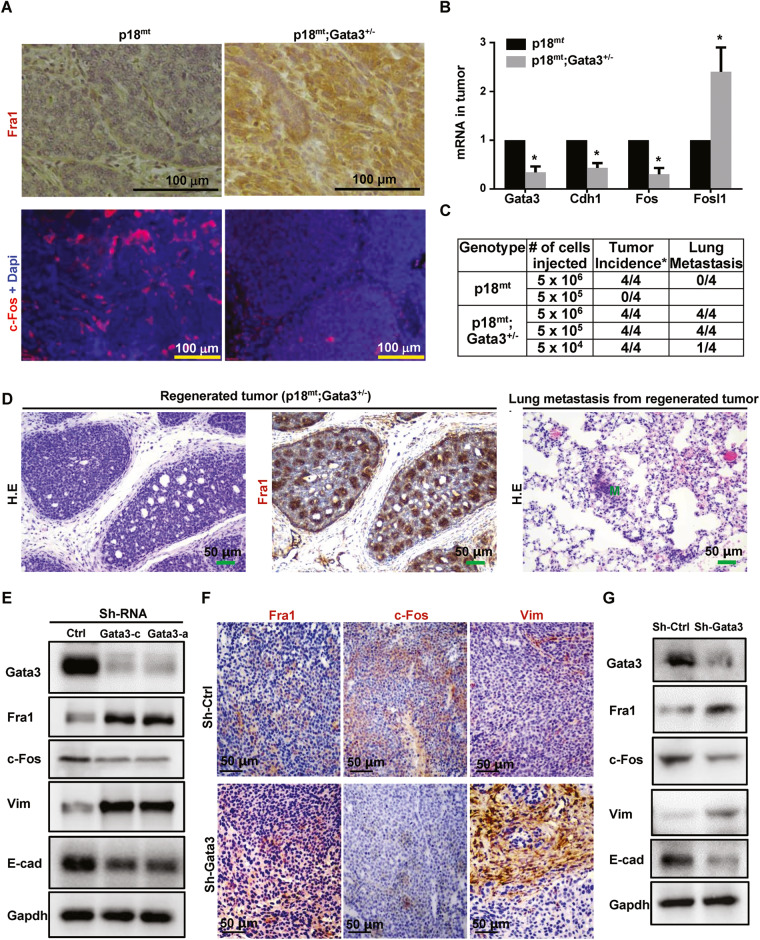
Depletion of Gata3 activates EMT with increase of Fra1 and decrease of c-Fos expression in mammary tumor development. **A** Representative IHC and IF analysis of primary mammary tumors with antibodies against Fra1 and c-Fos. **B** RNA extracted from representative mammary tumors were analyzed by qRT-PCR. Results represent the mean ± SD of three tumors from individual animal per group. The asterisk (*) denotes a statistical significance from p18^mt^;Gata3^+/−^ and p18^mt^ samples determined by unpaired T-test. **C** Primary tumor cells were transplanted into MFPs of NSG mice with estradiol supplement. Eight weeks later, recipient mice were dissected, regenerated mammary tumors and their lung metastasis were counted and analyzed. **D** Representative mammary tumors regenerated by p18^mt^;Gata3^+/−^ tumor cells and their lung metastasis were analyzed by HE (left and right) and IHC (middle). M, metastasis. **E**–**G** MMTV-PyMT mammary tumor cells were infected with psi-LVRU6GP-empty (sh-Ctrl) or psi-LVRU6GP-Gata3 (sh-Gata3), and then analyzed by western blot (**E**). 1 × 10^6^ MMTV-PyMT-sh-Ctrl) and MMTV-PyMT-sh-Gata3 tumor cells were transplant into the left and right MFPs of three female NCG mice, respectively, in a pairwise manner. Tumors generated by sh-Ctrl and sh-Gata3 cells were analyzed by IHC (**F**) and western blot (**G**).

We previously demonstrated that p18 is a downstream target of GATA3 and restrains mammary luminal cell proliferation and tumorigenesis [20]. We have also shown that haploid loss of Gata3 in p18 deficient mice, i.e., p18^mt^ mice including p18^−/−^ and p18^+/−^ mice, results in metastatic basal-like mammary tumors with EMT features [15, 16]. Taking advantage of the spontaneously developed p18^mt^ luminal type tumors that are proficient for Gata3 [15, 20, 36] and p18^mt^;Gata3^+/−^ basal-like tumors that are deficient for Gata3 [15, 16], we determined the expression of EMT-inducing transcription factors (EMT-TFs) and the key members of the AP-1 transcription factor. We found that the expression of most EMT-TFs including Fra1 was clearly enhanced and that of c-Fos was reduced in p18^mt^;Gata3^+/−^ tumors when compared with p18^mt^ tumors. Furthermore, the number of EMT positive tumors was significantly higher in p18^mt^;Gata3^+/−^ tumors than in p18^mt^ tumors (Table 1, Figs. 1A, B, S1). Notably, 13% of p18^mt^ tumors and 59% of p18^mt^;Gata3^+/−^ tumors were positive for Fra1, and Fra1-positive tumors were also EMT positive. Five out of seventeen p18^mt^;Gata3^+/−^ tumors metastasized to the lung, whereas none of eight p18^mt^ tumors metastasized. as we previously described [15]. Furthermore, all p18^mt^;Gata3^+/−^ mammary tumors with metastasis were Fra1-positive (Table 1 and Fig. 1). However, due to the development of kidney cysts and consequential renal failure, as well as various types of tumors in other organs including lymphoma and sarcoma in p18^mt^;Gata3^+/−^ mice [15, 16, 37], we were unable to follow the mammary tumor formation and metastasis in aged mice. The incidence of mammary tumor metastasis in p18^mt^;Gata3^+/−^ mice was underestimated. Interestingly, 63% of p18^mt^ tumors and 12% of p18^mt^;Gata3^+/−^ tumors were positive for c-Fos and c-Fos were barely detectable in EMT positive p18^mt^;Gata3^+/−^ tumors (Table 1, Figs. 1A, S1A). These data suggest that Gata3 deficiency downregulates c-Fos expression, leading cells to lose their epithelial fate and up-regulates Fra1, promoting cells to acquire mesenchymal feature. We determined the tumor-initiating capacity of tumor cells and found that as low as 5 × 10^4^ of p18^mt^;Gata3^+/−^ cells were able to regenerate tumors, whereas as high as 5 × 10^5^ of p18^mt^ cells did not yield tumors (Fig. 1C). All four mice that received 5 × 10^6^ p18^mt^ tumor cell transplants produced tiny tumors (smaller than 100 mm^3^ in size) with no metastasis, while during the same time period, all eight mice that received 5 × 10^6^ and 5 × 10^5^ p18^mt^;Gata3^+/−^ tumor cell transplants developed huge tumors (larger than 1000 mm^3^ in size) with lung metastasis (Fig. 1C). IHC analysis confirmed that regenerated p18^mt^;Gata3^+/−^ tumors in mammary glands were positive for Fra1 (Fig. 1D). These data illustrate that haploid loss of Gata3 in breast cancer cells enhances the CSC population and its properties in tumor initiation and metastasis. In summary, these results indicate that haploid loss of Gata3 reduces the expression of c-Fos and enhances the expression of Fra1, which leads to the activation of EMT and drives CSC function during basal-like mammary tumorigenesis and metastasis.

**Table 1:** Characterization of mammary tumors in mutant mice.

Tumor	Genotype^a^	
	p18^mt b^	p18^mt^;Gata3^+/− c^
EMT+ mammary tumor^d^	2/8 (25%)	13/17 (77%)^g^
Fra1+ mammary tumor^e^	1/8 (13%)	10/17 (59%)^h^
Mammary tumor with metastasis^f^	0/8	5/17 (29%)^i^
c-Fos+ mammary tumor^e^	5/8 (63%)	2/17 (12%)^j^

^a^All mice were in Balb/c-B6 mixed background and were at 8–22 months of age.

^b^This group contains eight p18^*+/−*^ and nineteen p18^−*/*−^ mice.

^c^This group contains ten p18^+/−^;Gata3^+/−^ and twenty four p18^−/−^;Gata3^+/−^ mice.

^d^At least two EMT markers (decreased E-cad, increased Vim, Fn1, Sma, or CD29) or two EMT-TFs, which include Twist, Slug, Snail, Foxc1, and Foxc2, were detected in >2% tumor cells by IHC, as we previously reported (Bai, Cancer Res., 2014).

^e^Expression of Fra1 or c-Fos was detected in >2% tumor cells by IHC.

^f^Three mammary tumors metastasized to lung, and the other two mammary tumors metastasized to lung and liver. All five mammary tumors with metastasis are Fra1 positive.

^g^A significance from p18^mt^;Gata3^+/−^ and p18^mt^ tumors by a two-tailed Fisher’s exact test (*p* = 0.028).

^h^A significance from p18^mt^;Gata3^+/−^ and p18^mt^ tumors by a two-tailed Fisher’s exact test (*p* = 0.042). All Fra1+ tumors are EMT+ tumors.

^i^No significance from p18^mt^;Gata3^+/−^ and p18^mt^ tumors by a two-tailed Fisher’s exact test (*p* = 0.1399).

^j^A significance from p18^mt^;Gata3^+/−^ and p18^mt^ tumors by a two-tailed Fisher’s exact test (*p* = 0.017).

### Depletion of Gata3 in luminal tumor cells results in upregulation of Fra1 and downregulation of c-Fos in the activation of EMT during tumorigenesis

We previously demonstrated that deficiency of Gata3 converts luminal-type mammary tumor cells into basal-like tumor cells with EMT activation [15, 16]. To directly test if deficiency of Gata3 in luminal tumor cells regulates c-Fos and Fra1 expression in mammary tumor development, we take advantage of the MMTV-PyMT luminal type mammary tumor model system we established [15, 16]. We knocked down Gata3 in MMTV-PyMT tumor cells that were isolated and screened from MMTV-PyMT mammary tumors and were confirmed as Gata3 proficient (Gata3^+/+^) luminal type before and after transplantation into mammary fat pads (MFPs) of recipient mice [Fig. 1E, and details in [15, 16]]. We observed that depletion of Gata3 in tumor cells reduced the expression of c-Fos, ERα, and E-Cad but enhanced the expression of Fra1 and Vim (Figs. 1E, S1C). We transplanted MMTV-PyMT tumor cells into MFPs of mice and analyzed newly generated mammary tumors. IHC analysis revealed that the tumors generated by Gata3-depleted cells displayed significantly more Fra1- and Vim-positive cells and less c-Fos-positive cells than tumors produced by control cells (Figs. 1F, S1D). Consistently, western blot showed the increase of Fra1 and Vim and a decrease of c-Fos and E-cad in Gata3 deficient tumors relative to Gata3 proficient tumors (Fig. 1G). These data further confirm the preferential expression of c-Fos in luminal tumor cells and Fra1 in basal-like tumor cells. These results demonstrate that depletion of Gata3 in luminal tumor cells also stimulates Fra1 and suppresses c-Fos expression in the activation of EMT during tumorigenesis.

### Reconstitution of Gata3 in Gata3- or Brca1-deficient tumor cells restores the expression of c-Fos and suppresses Fra1, inhibiting EMT and tumorigenesis

**Fig. 2:**
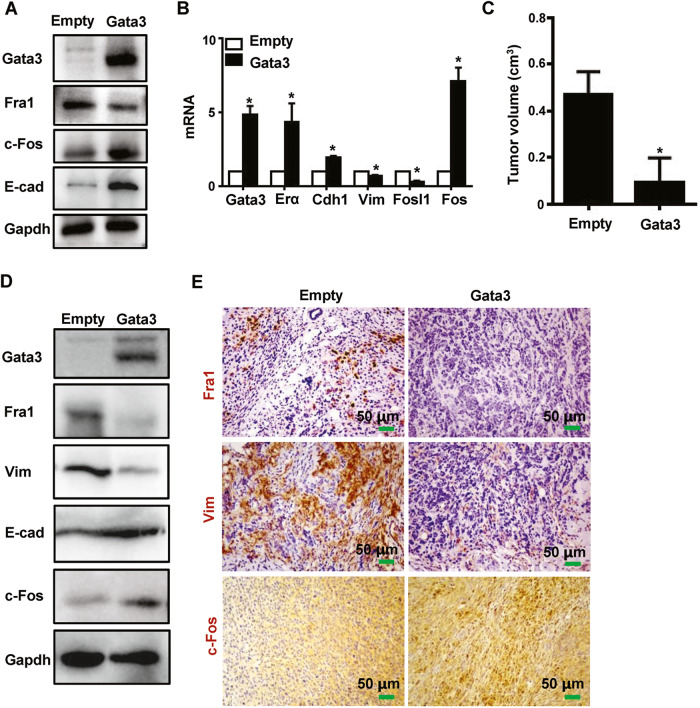
Reconstitution of Gata3 in Gata3-deficient tumor cells stimulates c-Fos and suppresses Fra1 expression in inhibition of EMT and mammary tumorigenesis. **A**, **B** p18^mt^;Gata3^+/−^ tumor cells were transfected with plvx-Flag (Empty) and plvx-Flag-Gata3 (Gata3), and then analyzed by western blot (**A**) and qRT-PCR (**B**). Data in (**B**) represent the mean ± SD from duplicates of two independent experiments. The asterisk (*) denotes a statistical significance from Empty and Gata3 samples determined by a two-tailed, paired T test. **C**, **E** p18^mt^;Gata3^+/−^ (1 × 10^6^) primary tumor cells infected with plvx-Flag (Empty) or plvx-Flag-Gata3 (Gata3) were transplanted into the left and right MFPs of female NCG mice. Tumors regenerated were analyzed by their size (**C**), western blot (**D**), and IHC (**E**). Data in (**C**) represent the average tumor volumes ± SD of three tumors from individual animals per group. *P* < 0.05 represent statistical significance from GATA3 and Empty tumors determined by a two-tailed, paired T test.

We screened and characterized ten tumor cell lines from ten individual p18^mt^;Gata3^+/−^ primary mammary tumors and established a Gata3 deficient basal-like mammary tumor model system [15, 16]. We then tested whether reconstitution of Gata3 could restore AP-1 expression and epithelial features of Gata3 deficient tumor cells. As expected, we found that reconstitution of Gata3 significantly reduced the expression of Fra1 and Vim but stimulated the expression of c-Fos, ERα, and E-cad (Fig. 2A, B). This suggests that reconstitution of Gata3 restores the expression of c-Fos and other epithelial genes to gain epithelial features and concurrently inhibits the expression of Fra1 to suppress mesenchymal traits, leading to the activation of mesenchymal–epithelial transition (MET) in vitro. We infected p18^mt^;Gata3^+/−^ tumor cells with pLvx-Flag (Empty) and pLvx-Flag-Gata3 (Gata3), respectively, and established Empty and Gata3 stably expressing cells. These cells were then transplanted into the MFP of NCG mice. We observed that Gata3-expressing cells produced significantly smaller tumors than Empty-expressing cells (Fig. 2C). Relative to Empty-tumors, Gata3-tumors expressed drastically less Fra1 and Vim but more c-Fos and E-cad (Fig. 2D, E). Notably, Gata3-tumors displayed less heterogeneous morphology in cell shape and more glandular structures than Empty-tumors (Fig. 2E), indicating that Gata3-tumors are well-differentiated relative to Flag-tumors. These results confirm that reconstitution of Gata3 restores c-Fos and suppresses Fra1 expression in the activation of MET and inhibition of Gata3 deficient tumor initiation.

Inspired by our finding that GATA3 functions downstream of BRCA1 to suppress EMT in breast cancer [15] and that BRCA1 deficiency stimulates the expression of FRA1 in mammary tumors [38, 39], we hypothesized that reconstitution of GATA3 in BRCA1 deficient tumor cells also restores the expression of AP-1, thus inhibiting tumorigenesis. First, we confirmed the decrease of c-Fos and increase of Fra1 in p18^mt^;Gata3^+/−^ and p18^mt^;Brca1^+/−^ tumors when compared with those in p18^mt^ tumors (Fig. S1). Notably, the expression level of c-Fos and Fra1 in p18^mt^;Brca1^+/−^ and p18^mt^;Gata3^+/−^ tumors were comparable (Fig. S1). Next, we generated empty- and Gata3-expressing p18^mt^;Brca1^+/−^ tumor cells, as previously reported [15], and performed similar analysis as for p18^mt^;Gata3^+/−^ tumor cells. We found that Gata3-expressing cells generated smaller tumors with less Fra1 and Vim but more c-Fos and E-Cad than tumors generated by control cells [Fig. S2, and details in [15]]. These data suggest that Gata3 functions downstream of Brca1 to promote c-Fos and suppress Fra1 expression in inhibition of EMT and mammary tumor initiation.

### GATA3 suppresses FRA1 and promotes c-FOS expression in inhibition of EMT and tumorigenesis of human breast cancer cells

**Fig. 3:**
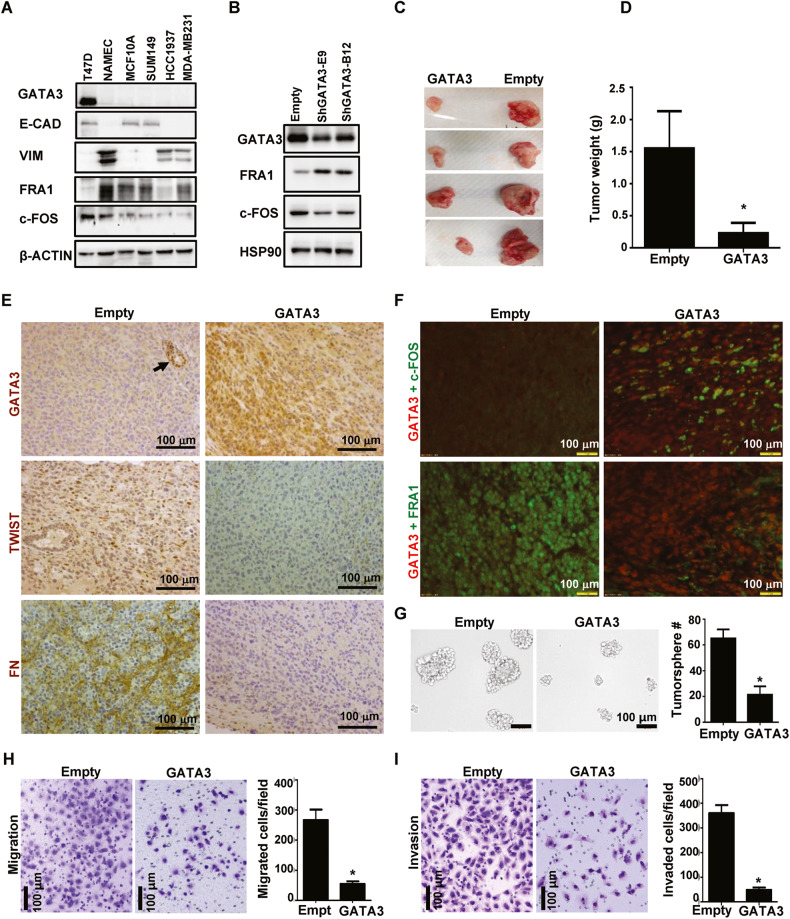
GATA3 positively regulates c-FOS and negatively regulates FRA1 in inhibition of tumorigenesis and tumorsphere forming potential of human breast cancer cells. **A** Analysis of human breast cancer cell lines, NAMEC, and basal epithelial cell line MCF10A. **B** T47D were infected with pGIPZ-empty (Empty) or pGIPZ-shGATA3 targeting different sequences of human GATA3 (shGATA3-E9 and shGATA3-B12), selected with puromycin, and analyzed by western blot. **C**–**F** MDA-MB231 were infected with pBabe-puro-empty (Empty) and pBabe-puro-GATA3 (GATA3), selected with puromycin, and transplanted into the MFPs of female NSG mice. Gross appearance (**C**) and weight (**D**) of the tumors generated are shown. Tumors were analyzed by IHC (**E**) and IF (**F**). Data in (**D**) represent the mean ± SD for tumors in each group (*n* = 4). *P* < 0.05 represent statistical significance from GATA3 and empty tumor determined by a two-tailed, paired T test. A GATA3-positive mammary gland of the recipient mice is indicated by black arrow in (**E**). **G** 10^3^ MDA-MB231-empty and MDA-MB231-GATA3 cells were cultured to generate primary in 10 days. The number of spheres larger than 100 μm was quantified from triplicate experiments. The results represent the mean ± SD of three independent experiments. Statistical significance was determined by a two-tailed, unpaired T test. **H**, **I** 2 × 10^4^ MDA-MB231-empty and MDA-MB231-GATA3 cells were seeded into the upper chamber of the Migration chambers (**H**) or Invasion chambers (**I**). 24 (**H**) or 48 (**I**) hours later, the cells on the lower surface of the membrane were fixed, stained, and counted. The results represent the mean ± SD of three independent experiments. Statistical significance was determined by a two-tailed, unpaired T test.

We analyzed a panel of human breast cell lines, including non-adherent mammary epithelial cell (NAMEC), a naturally arising mesenchymal cell line from HMLE cells that had spontaneously undergone an EMT. We found that two widely used luminal cell lines, T47D and MCF7, expressed high levels of GATA3 and c-FOS but low levels of FRA1. In contrast, basal and mesenchymal cell lines, including SUM149, HCC1937, MDA-MB-231, NAMEC, and MCF10A, expressed high levels of FRA1 and low levels of GATA3 and c-FOS (Figs. 3A, S3A, B). Knockdown of GATA3 enhanced the expression of FRA1 but reduced the expression of c-FOS in T47D cells (Fig. 3B). We generated stable GATA3-expressing MDA-MB-231 cells and transplanted them into MFPs of NCG mice. Consistent with the data obtained by reconstitution of Gata3 in mouse cells, GATA3-expressing MDA-MB-231 cells produced significantly smaller tumors than control MDA-MB-231 cells (Fig. 3C, D), and GATA3-expressing tumors expressed more c-FOS but less FRA1, TWIST, and FN than control tumors (Fig. 3E, F). Furthermore, we found that GATA3-expressing MDA-MB-231 cells generated significantly less tumorspheres and displayed significantly less motility and invasion than control MDA-MB-231 cells (Figs. 3G–I, S3C). These results confirm that reconstitution of GATA3 in human breast cancer cells regulates AP-1 expression inhibiting EMT, tumorsphere forming potential, motility, invasion, and tumorigenesis.

### GATA3 binds to the FOSL1 and FOS loci to regulate their transcription

**Fig. 4:**
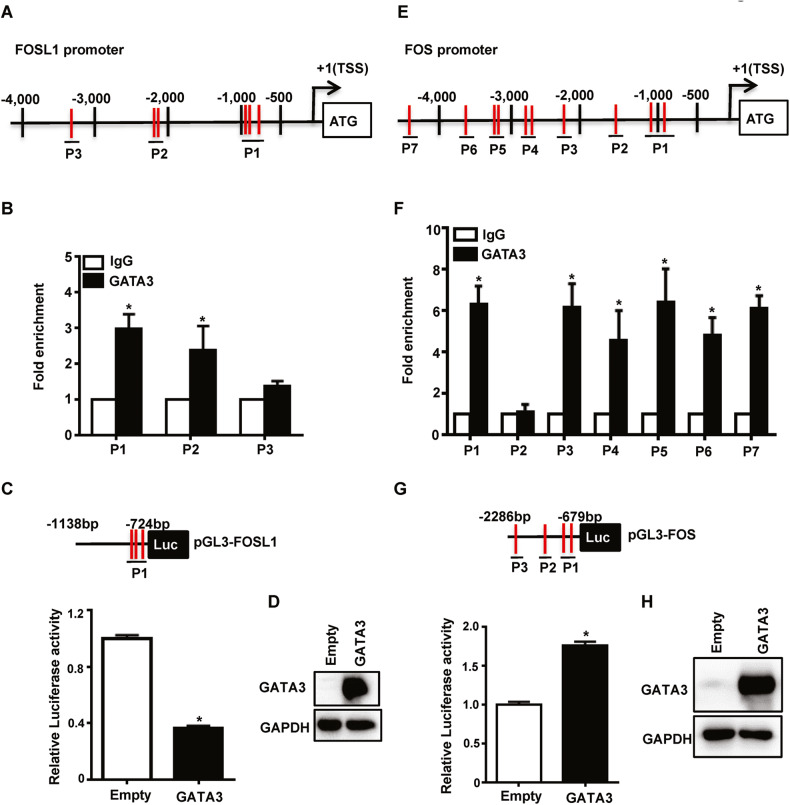
GATA3 binds to FOSL1 and FOS loci to regulates their transcription. **A**, **E** Diagram showing the location of putative GATA3 binding sites (red bars) in human FOSL1 gene (**A**) and FOS gene (**E**). +1, transcription start site (TSS). Locations of the primer pairs used in ChIP analysis are shown. **B**, **F** ChIP analysis of endogenous GATA3 binding to the FOSL1 locus (**B**) and FOS locus (**F**) in T47D cells. The results were normalized to the amount of input and compared with the IgG-negative controls. Data are represented as mean ± SD. The asterisk (*) denotes a statistical significance from IgG and anti-GATA3 immunoprecipitated samples by a two-tailed, unpaired T test. **C**, **G** MDA-MB231 cells were transduced with pLvx-Flag (Empty) and pLvx-Flag-GATA3 (GATA3) to generate empty- and GATA3-expressing stable cells, which were then transfected with Renilla, pGL3-basic, and pGL3-FOSL1 (**C**) or pGL3-FOS (**G**). Cell lysates were then collected and assayed for pGL3-FOSL1 (**C**) and pGL3-FOS (**G**) luciferase activities. Data represent the mean ± SD from duplicates of two independent experiments. The asterisk (*) denotes a statistical significance from Empty and GATA3 samples determined by a two-tailed, paired T test. **D**, **H** Protein lysates from (**C**) and (**G**) was analyzed by western blot.

To determine whether the transcription factor GATA3 directly regulates FRA1 expression at the transcriptional level, we performed bioinformatic analysis and found at least six putative GATA3 binding sites in the FOSL1 gene promoter (Fig. 4A). Using three pairs of primers that cover all six putative GATA3 binding sites, we performed ChIP assays and found that two out of three amplicons (P1, P2) covering five GATA3 binding sites in T47D cells were specifically enriched in the immunoprecipitation of GATA3 (Fig. 4B). We generated FRA1 promoter-luciferase fusion plasmids, pGL3-FOSL1, containing a promoter region that covers P1 with three GATA3 binding sites (Fig. 4C). We transduced MDA-MB231 cells with pLvx-3xFlag (Empty) and pLvx-3xFlag-GATA3 (GATA3) and established empty- and GATA3-expressing stable cells (Fig. 4D). We transfected pGL3-FOSL1 and renilla plasmids into the empty- and GATA3-expressing cells and observed that ectopic GATA3 drastically reduced the activity of pGL3-FOSL1 promoter by ~52% relative to an empty control (Fig. 4C). These results indicate that GATA3 directly binds to the FOSL1 locus to repress its transcription.

Similarly, we also performed bioinformatic analysis and found that there exist at least ten putative GATA3 binding sites in the FOS gene promoter (Fig. 4E). We performed ChIP assays using seven pairs of primers that cover all ten putative GATA3 binding sites. Six out of seven amplicons (P1, P3, P4, P5, P6, P7) were specifically enriched in the immunoprecipitation of GATA3 (Fig. 4F). We also generated FOS promoter-luciferase fusion plasmids, pGL3-FOS, containing a promoter region that covers P1, P2, and P3 with four GATA3 binding sites. We transfected pGL3-FOS and renilla plasmids into empty- and GATA3-expressing cells and found that relative to an empty control, ectopic GATA3 significantly enhanced the activity of pGL3-FOS promoter by 1.7 folds (Fig. 4G, H). These data confirm that GATA3 binds to the FOS promoter to activate its transcription.

### Targeted deletion of Fra1 in Gata3-deficient tumor cells inhibits EMT, preventing tumorigenesis and metastasis

**Fig. 5:**
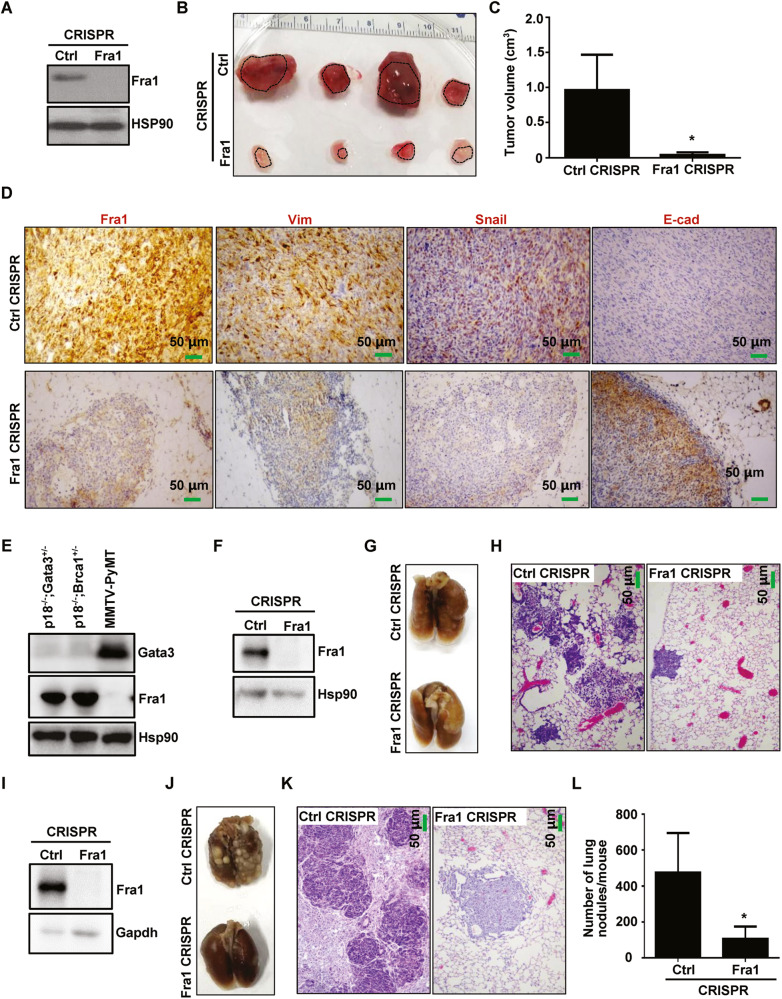
Knockout of Fra1 in Gata3-deficient tumor cells inhibits EMT, tumorigenesis, and metastasis. **A** p18^mt^;Gata3^+/−^ tumor cell were transfected with Fra1 (Fra1 CRISPR) and Control (Ctrl CRISPR) Double Nickase plasmids then selected with puromycin for 3 days. Fra1- and control-knockout cells were then analyzed by western blot. **B**–**D** 1 × 10^4^ Fra1- and Ctrl-knockout p18^mt^;Gata3^+/−^ mammary tumor cells were inoculated into the left and right inguinal MFPs of NSG mice, respectively, in a pairwise manner. Four months after transplantation, mice were dissected. Gross appearance (**B**) and volume (**C**) of the regenerated tumors were determined. Tumors regenerated were analyzed by IHC (**D**). Data in (**C**) represent the mean ± SD of four tumors in each group. The asterisks (*) denote a statistical significance from Fra1- and Ctrl-knockout tumors determined by a two-tailed, paired T test. **E** Confirmation the expression of Gata3 and Fra1 in p18^mt^;Gata3^+/−^ and p18^mt^;Brca1^+/−^ mammary tumor cells. (**F**) Fra1- and Ctrl-knockout p18^mt^;Gata3^+/−^ mammary tumor cells were analyzed before tail vein injection. **G**, **H** 1 × 10^6^ Fra1- and Ctrl-knockout p18^mt^;Gata3^+/−^ mammary tumor cells were injected via the tail vein into NCG mice. Four weeks after tail vein injection, the lungs were removed and examined for gross appearance (**G**) and H.E staining (**H**). **I** Fra1- and Ctrl-knockout p18^mt^;Brca1^+/−^ mammary tumor cells were analyzed before tail vein injection. **J**, **K** 1 × 10^6^ Fra1- and Ctrl-knockout p18^mt^;Brca1^+/−^ mammary tumor cells were injected via the tail vein into NCG mice. Four weeks after tail vein injection, the lungs were removed and examined for gross appearance (**J**) and H.E staining (**K**). **L** Quantification of the number of metastatic nodules in the lungs in (**G**) and (**J**). Data represent the mean ± SD for the numbers of metastatic nodules detected in all lobes of the lungs in each group (*n* = 5). Ctrl CRISPR group includes two mice injected with p18^mt^;Gata3^+/−^-Ctrl CRISPR cells and three mice injected with p18^mt^;Brca1^+/−^-Ctrl CRISPR cells. Fra1 CRISPR group includes two mice injected with p18^mt^;Gata3^+/−^-Fra1 CRISPR cells and three mice injected with p18^mt^;Brca1^+/−^-Fra1 CRISPR cells. Statistical significance was determined by a two-tailed, unpaired T test.

We knocked out Fra1 in p18^mt^;Gata3^+/−^ tumor cells and observed that Fra1 knockout drastically reduced the expression of the EMT markers Vim, Snail, Twist, Slug (Figs. 5A, S4A, B). We transplanted these tumor cells into mice and although all mice received 1 × 10^4^ Fra1- and control-knockout p18^mt^;Gata3^+/−^ tumor cells, tumors generated by Fra1-knockout cells were tiny and significantly smaller than those generated by control cells (Fig. 5B, C). Tumors generated by Fra1-knockout cells were less heterogeneous with a smaller mitotic index, were less invasive, and had more glandular structure, compared with tumors generated by control cells (Fig. S4C). These findings suggest that knocking out Fra1 in Gata3 deficient tumor cells leads to development of relatively well-differentiated tumors. Notably, tumors generated by Fra1-knockout cells exhibited significantly reduced levels of Vim and Snail, but enhanced level of E-cad, compared to tumors generated by control cells (Fig. 5D). Moreover, the knockout of Fra1 or reconstitution of Gata3 in Gata3 deficient tumor cells produced a very similar phenocopy in terms of reducing mesenchymal traits and inducing epithelial features in tumor suppression (Figs. 5 and 2, S2). Taken together, these results demonstrate that Fra1 is required for the activation of EMT in Gata3-deficient tumorigenesis.

Taking advantage of Gata3 and Brca1 deficient tumor cells lacking Gata3 and expressing high level of Fra1 [15, 38, 39] (Fig. 5E), we knocked out Fra1 in these cells by using the CRISPR/Cas9 system (Fig. 5F, I). We injected these cells into the mice through tail vein and found that lung nodules generated by Fra1-knockout cells were significantly less than those generated by control cells (Fig. 5G, H, J–L). These data suggest that knockout of Fra1 in Gata3 deficient tumor cells inhibits tumor metastasis.

### Reconstitution of c-Fos in Gata3 deficient tumor cells fails to restore epithelial features during tumorigenesis

**Fig. 6:**
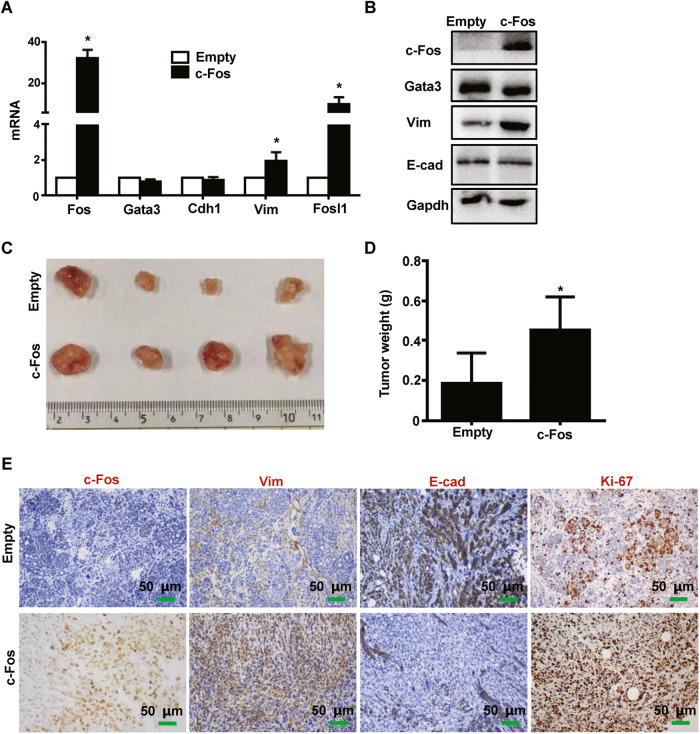
Reconstitution of c-Fos in Gata3-deficient tumor cells stimulates FRA1 expression and promotes tumorigenesis. **A**, **B** p18^mt^;Gata3^+/−^ tumor cell were infected with pEZ-Lv201 (Empty) and pEZ-Lv201-c-Fos (c-Fos), selected with puromycin, and analyzed by qRT-PCR (**A**) and western blot (**B**). The asterisk (*) denotes a statistical significance from empty and c-Fos samples determined by a two-tailed, paired T test. **C**, **D** p18^mt^;Gata3^+/−^ tumor cell infected with pEZ-Lv201 (Empty) and pEZ-Lv201-c-Fos (c-Fos) were transplanted into the left (Empty) and right (c-Fos) MFPs of female NCG mice. Gross appearance (**C**) and weight (**D**) of the tumors generated were determined. Data in (**D**) represent the mean ± SD of four tumors in each group. The asterisks (*) denote a statistical significance from c-Fos and Empty tumors determined by a two-tailed, paired T test. **E** Tumor generated from (**C**) was analyzed by IHC.

We transduced two independent p18^mt^;Gata3^+/−^ tumor cell lines with pEZ-Lv201 (Empty) and pEZ-Lv201-c-Fos (c-Fos), thereby establishing empty- and c-Fos-expressing stable cells. Surprisingly, we noticed that reconstitution of c-Fos did not enhance the expression of E-cad as expected, but rather induced the expression of Vim and Fra1 (Figs. 6A, B, S5A, B). However, this result is consistent with the previous discovery that FOSL1 can be directly induced by c-FOS [32, 40]. We then transplanted these tumor cells into mice. In contrast to our expectation, tumors generated by c-Fos-expressing cells were significantly larger than tumors generated by control cells (Figs. 6C, D, S5C). IHC analysis revealed that Ki67-positive cells in c-Fos-expressing tumors were in a statistically higher ratio than the Ki67-positive cells in control tumors (Fig. 6E), confirming that c-Fos is an oncogene and promotes cell proliferation. Notably, the expression of Vim and E-cad displayed a drastic inter- and intra-tumor heterogeneity in c-Fos-expressing tumors (Figs. 6E, S5D), which may reflect the unique situation that the tumor cells highly, but heterogeneously expressing both c-Fos and Fra1, the former of which activated E-cad, and the latter of which enhanced Vim. These results suggest that downregulation of c-Fos plays an important role in the activation of EMT by Gata3 deficiency; however, reconstitution of c-Fos is not sufficient to restore luminal and/or epithelial traits of Gata3 deficient tumor cells that have undergone EMT.

### The expression of GATA3 is correlated with that of FRA1 and c-FOS in human breast cancers

**Fig. 7:**
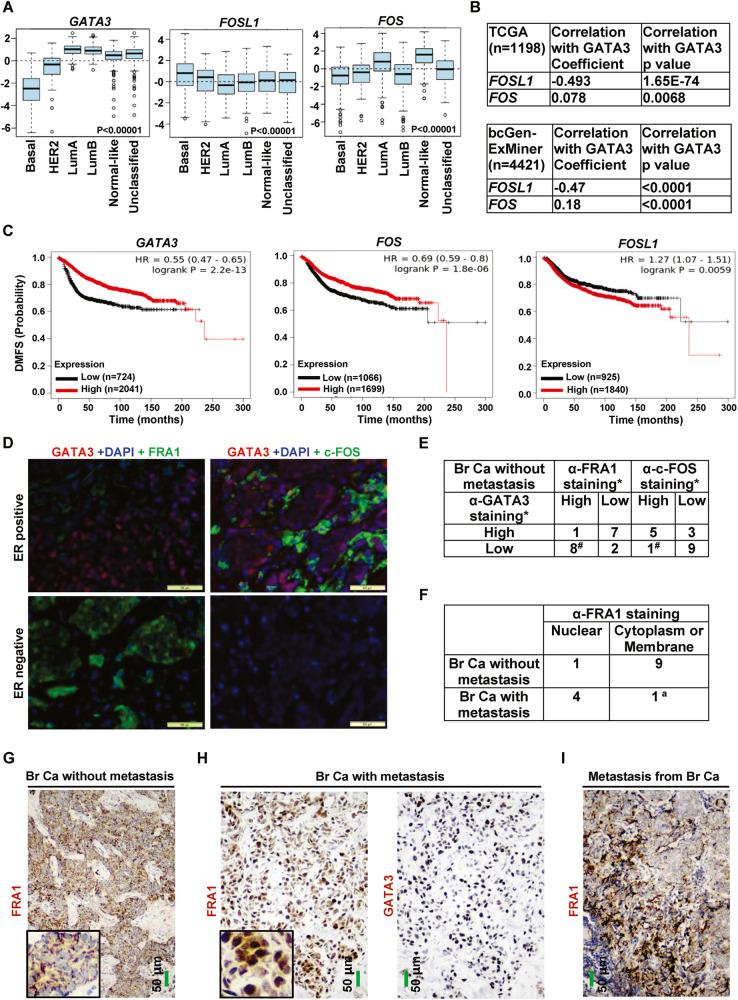
Correlation analysis of FRA1 and c-FOS with GATA3 in human breast cancers. **A** Analysis of gene expression in GOBO breast cancer database according to molecular subtype. LumA, luminal A; LumB, luminal B. *p* < 0.00001 represent statistical significance from different subtypes. **B** Correlation analysis of the expression of GATA3 and FOSL1 or FOS for TCGA and bcGenExMiner v4.8 breast cancer patients. **C** Kaplan–Meier plots of the distant metastasis-free survival (DMFS) of breast cancer patients. Patient groups were separated based on GATA3, FOSL1, or FOS mRNA level. **D** Representative IF analysis of human breast cancer samples with antibodies against GATA3, FRA1, and c-FOS. **E** Summary of the immunostaining analysis for human breast cancer samples. *, “High” expression represents the samples positively stained with an antibody in more than 2% cells (i.e., scores equal to or higher than “+” in Fig. S6A). “Low” expression represents the samples negative or positively stained with an antibody in less than 2% cells (i.e., scores less than “+/−” in Fig. S6A). ^#^, a significance from GATA3 high and GATA3 low tumors by a two-tailed Fisher’s exact test. Br Ca, Breast Cancer. **F** Summary of the immunostaining analysis for human breast cancers with or without lymph node metastasis. “Nuclear” represents that more than half of the FRA1 positive tumor cells displayed predominant nuclear FRA1 staining, and “Cytoplasm and Membrane” represents that more than half of the FRA1 positive tumor cells stained with anti-FRA1 predominantly in cytoplasm and membrane. The asterisk (^a^) denotes a significance from breast cancers with metastasis and breast cancers without metastasis by two-tailed Fisher’s exact test. **G**–**I** Representative IHC analysis of human breast cancers without metastasis (**G**), breast cancers with lymph node metastasis (**H**), and lymph node metastasis from breast cancers (**I**). Sections in (**H**) were two serial sections from a breast cancer with metastasis.

Gene-expression profiling analyses have categorized human breast tumors into five intrinsic subtypes: basal-like, Her2+, luminal A, luminal B, and normal breast-like [23]. Among these subtypes of breast cancer, the basal-like subtype is characterized by the low to absent expression of luminal differentiation markers including GATA3 and high enrichment for EMT markers. In contrast, luminal A and B subtypes are characterized by the high levels of luminal and epithelial markers and low enrichment for EMT markers. To determine whether our mouse genetic analysis models human breast cancers, we queried the expression of GATA3 and AP-1 in breast cancer patient sample sets. We found that expressions of GATA3, FOS, and FOSL1 were highly correlated with intrinsic subtypes (Fig. 7A). Specifically, the mRNA levels of GATA3 and FOS were low, whereas the mRNA levels of FOSL1 were high in the ER-negative, basal-like subtype. In the ER-positive luminal A subtype, the mRNA levels of GATA3 and FOS were high and the mRNA levels of FOSL1 were low (Figs. 7A, S6C, D). Pearson correlation analysis revealed a statistically significant inverse correlation between GATA3 with FOSL1 mRNA levels. Although not as strong as the correlation between GATA3 and FOSL1, a positive correlation between GATA3 and FOS mRNA levels was also detected in human breast cancers (Figs. 7B, S6B). These findings are consistent with our observations in mouse models.

Kaplan–Meier analysis of distant metastasis-free survival (DMFS) revealed that the expression of GATA3, FOS, and FOSL1 genes was significantly predictive of patient outcome. High FOSL1 expression predicted a poor patient outcome, and low GATA3 or FOS expression also predicted poor patient outcomes (Fig. 7C). This shows that human basal-like tumors are characterized by low GATA3, low FOS and high FOSL1. We then determined whether the expression of these genes was able to predict DMFS for patients with different subtypes of breast cancer and patients with or without lymph node metastasis. We noticed that a low level of GATA3 and FOS mRNA predicted poor outcomes for patients with luminal A or B and basal-like subtype breast cancers, and high FOSL1 expression also predicted a poor outcome for patients with luminal A subtype breast cancers (Fig. S7A). We failed to detect a significance of FOSL1 mRNA in the prediction of survival for basal-like patients, which may be partially explained by the upregulation or activation of FOSL1 or FRA1 at both transcriptional and post-transcriptional levels in the development and progression of basal-like breast cancer [15, 35]. Therefore, upregulation of FOSL1 mRNA alone may not be sufficient to predict survival for basal-like breast cancer. Interestingly, we observed that high FOS expression predicted a poor outcome for patients with HER2+ subtype breast cancer, which requires further investigation and interpretation (Fig. S7A). In consistent with above findings, we also found that low GATA3 mRNA predicted poor DMFS for patients with or without lymph node metastasis and that high FOSL1 predicted a poor outcome for patients with no lymph node metastasis. However, we failed to detect a significant association between FOSL1 mRNA and prediction of survival for patients with lymph node metastasis (Fig. S7B).

To test whether these observations are consistent at the protein level, we took advantage of our previously published resource of 43 invasive breast cancers with no metastasis [36]. We performed immunostaining analysis of 8 ER+ and 10 ER− primary human breast cancers. We found that FRA1 was readily detected in ER− and GATA3 weak or non-detectable tumor cells, but hardly detectable in ER+ and GATA3+ tumor cells (Figs. 7D, S6A, S8). Conversely, c-FOS was readily detected in ER+ and GATA3+ tumor cells, but hardly detectable or weakly expressed in ER− and GATA3 weak or non-detectable tumor cells (Figs. 7D, S6A, S8). Further analysis revealed that FRA1 and c-FOS detected by immunostaining were inversely and positively correlated with GATA3, respectively (Figs. 7E, S6A). Together, these clinical findings are consistent with our results in mice, suggesting an opportunity to use murine systems to further explore how GATA3 regulates FRA1 and c-FOS to control human breast biology as well as cancer development and progression.

We then carefully examined 10 breast cancers without metastasis and 5 paired breast cancers with lymph node metastasis. IHC analysis revealed that 9 out of 10 breast cancers without metastasis predominantly expressed FRA1 in the cell membrane and cytoplasm, which was consistent with the findings derived from IF analysis (Figs. 7D, G, and S9A). Notably, 4 out of 5 breast cancers with lymph node metastasis displayed a predominant nuclear staining, which was significantly different from the expression pattern of FRA1 in cancers without metastasis (Figs. 7F, G, H, and S9A, B). Again, the expression of FRA1 in these cancers was also negatively correlated with that of GATA3 (Figs. 7H and S9B). These results suggest that, unlike FRA1 in breast cancers without metastasis, FRA1 in most breast cancers with metastasis is likely activated and that the activation of FRA1 is, at least, partially, induced by GATA3 deficiency. In addition, we also observed high FRA1 expression in lymph node metastasis derived from breast cancers (Figs. 7I and S9C). Together, these findings are in line with our discovery in mice that loss of Gata3 activates Fra1, promoting mammary tumor metastasis.

## Discussion

In the present study, we have demonstrated that heterozygous germline deletion of Gata3 up-regulates Fra1, downregulates c-Fos, activates EMT, and enhances mammary tumor initiating and metastatic potential. Depletion of Gata3 in luminal tumor cells also up- and downregulates Fra1 and c-Fos, respectively, leading to the activation of EMT and promotion of tumorigenesis. Consistently, reconstitution of Gata3 in Gata3- or Brca1-deficient human and mouse mammary tumor cells restores the expression of c-Fos and suppresses Fra1 in inhibiting EMT, motility, invasion, and tumorigenesis. We discovered that GATA3 binds to the FOSL1 locus to repress its transcription and, notably, to the FOS locus to activate its transcription. Deletion of Fra1, but not reconstitution of c-Fos, in Gata3-deficient tumor cells inhibits EMT suppressing tumorigenesis and metastasis. This suggests that in GATA3-deficient tumor cells, the acquisition of mesenchymal traits by the activation of Fra1 is dominant over the loss of epithelial features by the deficiency of c-Fos, in activating EMT and driving CSC function. Consistent with the findings derived from mouse models, we found that in human breast cancers, GATA3 expression is negatively correlated with FRA1 and positively correlated with c-FOS. Specifically, GATA3 and c-FOS are low, and FRA1 is high in basal-like subtype, whereas GATA3 and c-FOS are high and FRA1 is low in luminal A subtype. Together, our results indicate that in mammary tumor cells GATA3 directly activates the transcription of FOS to maintain their luminal and epithelial features while concurrently repressing the transcription of FOSL1 to suppress aberrant mesenchymal differentiation, i.e., inhibiting EMT. We also demonstrated that FRA1 is required for the activation of EMT during GATA3 deficient tumor initiation and metastasis.

It has been demonstrated that c-FOS is preferentially expressed in mammary epithelial cell lines and non-CSCs, whereas FRA1 is specifically detected in CSCs and mammary epithelial cells that have undergone EMT. c-FOS binds to and activates genes encoding E-cadherin and Crumb3, two key epithelial proteins, to maintain epithelial features of non-CSCs. FRA1 transactivates most, if not all, EMT-TFs and interacts with several intrinsic and extrinsic pathways to sustain or promote mesenchymal traits and drive CSC-like function [32, 33, 35]. During the activation of EMT program, there is a switch from the use of c-FOS to FRA1 as the preferred component of AP-1 transcription factor complexes [35]. These findings suggest that loss of c-FOS-maintained epithelial features may collaborate with the acquisition of FRA1-induced mesenchymal traits to activate EMT and drive CSC function. Although it has been reported that a few transcription factors including Myc, p53, AP-1, SNAIL, and TWIST trans-activate FOSL1 [32, 33], it remains unknown whether a transcription factor trans-represses FOSL1 and whether the transcription factor concurrently transactivates FOS in the regulation of EMT during tumorigenesis. The function of GATA3 in suppressing EMT and metastasis in breast cancers has been well studied in cell line models [26, 27, 41, 42] in which overexpression of GATA3 inhibits the expression of some of the EMT-TFs and enhances the expression of E-cad. However, due to the proliferative defects or apoptosis induced by loss of Gata3 in mammary epithelial and tumor cells [20, 28, 43], it remains elusive whether and how loss of function of Gata3 regulates EMT in breast cancer development and progression. Taking advantage of p18;Gata3 double mutant mouse models, in which depletion of Gata3 converts p18 deficient luminal tumors into BLBCs with EMT features and enrichment of CSC characteristics [15, 16], we demonstrated that deficiency of Gata3 activates Fosl1 transcription and concurrently represses Fos transcription in the activation of EMT, driving tumor initiation and metastasis. Notably, deletion of Fra1, like reconstitution of Gata3, in Gata3 deficient tumor cells inhibits EMT, preventing tumorigenesis and/or metastasis, whereas reconstitution of c-Fos in Gata3 deficient cells fails to inhibits EMT during tumorigenesis. These data provide genetic and biochemical evidence indicating that loss of function of Gata3 in mammary tumor cells activates Fosl1 to promote mesenchymal traits and CSC function and concurrently represses Fos to lose epithelial features. Furthermore, our findings demonstrate that the activation of Fosl1 is dominant over the repression of Fos in GATA3 deficiency-induced EMT during tumorigenesis.

FOSL1 is regulated at both the transcriptional and post-transcriptional levels [32, 33]. Phosphorylated FRA1 associates with members of the JUN family of transcription factors to form heterodimeric AP-1 complexes to transcriptionally regulate target gene expression [32, 34, 35]. The activation of EMT and the formation of CSCs from non-stem cells involve a shift from EGFR to PDGFR signaling, resulting in the PKCα-dependent activation of FRA1 [35]. In the previous studies, we and others have shown that GATA3 recruits BRCA1 to its binding sites in the promoters of FOXC1/2, TWIST, and TGFβR2 genes to repress their transcription [36, 39, 44]. We uncovered that BRCA1 binds to the GATA3 binding sites in the promoter of PDGFRβ to repress its transcription and that depletion of Brca1 stimulates the expression of PDGFRβ activating the PDGFRβ-PKCα-FRA1 pathway to induce EMT and drive CSC function in breast cancer [38]. We also demonstrated that GATA3 functions downstream of BRCA1 to suppress EMT in breast cancer [15]. Taking into consideration our findings in this and previous studies that Brca1 deficiency stimulates Fra1 expression and reconstitution of Gata3 restores suppression of Fra1 in Brca1 deficient tumor cell during inhibition of EMT and tumorigenesis [38], we propose that the BRCA1-GATA3 axis represses FOSL1 at both transcriptional and post-transcriptional levels to doubly strengthen the inhibitory effect of BRCA1 and GATA3 in the regulation of FRA1 and FRA1-mediated EMT. The BRCA1 and GATA3 complex, at the transcription level, binds to FOSL1 locus repressing its expression. BRCA1 and GATA3, at post-transcriptional level, inhibit PDGFRβ-PKCα signaling pathway to block phosphorylation of FRA1, abolishing its association with the JUN family of transcription factors, thereby preventing the transactivation of target gene expression. Accordingly, we propose that PDGFRβ-PKCα-FRA1 pathway is a potential therapeutic target for GATA3-deficient BLBCs.

Though AP-1 family proteins have long been recognized as oncoproteins [34], it is poorly understood whether c-FOS controls tumor cell fate and how c-FOS is regulated in the activation of EMT during mammary tumorigenesis. c-FOS has been reported to be mainly expressed in epithelial cells. It activates the transcription of CDH1 (encoding E-cadherin) and plays a critical role in maintaining the state of the epithelial cells [35]. In this study, we found that in both human and mouse breast cancers GATA3 and c-FOS are preferentially expressed in luminal-type mammary tumor cells, and are very low or absent in BLBCs. Depletion of Gata3 downregulates c-Fos, activates EMT and enhances tumor-initiating potential. Reconstitution of Gata3 in Gata3 deficient tumor cells restores c-Fos expression inhibiting EMT and tumorigenesis. However, reconstitution of c-Fos fails to restore epithelial features in Gata3 deficient tumor cells. These results suggest that depletion of Gata3 suppresses the expression of c-Fos, which is, at least partially responsible for the loss of luminal and/or epithelial cell features of the tumor cells, but reconstitution of c-Fos is not sufficient to restore luminal and/or epithelial traits of Gata3 deficient tumor cells that have undergone EMT. Notably, we discovered that reconstitution of c-Fos in Gata3-deficient tumor cells promotes EMT and tumor cell growth. Given the finding that c-FOS also transactivates FOSL1 expression [32, 40] and functions as an oncogene to promote tumor cell growth [34], it is not surprising that overexpression of c-FOS promotes EMT and accelerates tumor development and progression.

## Materials and methods

### Cell culture, overexpression and knockdown, and tumorsphere formation assay

MCF-7, T47D, MDA-MB231, MCF10A, HMLE (ATCC), SUM149 (Dr. Sendurai Mani, University of Texas, Houston, TX), and HCC1937 (Dr. Jennifer Hu, University of Miami, Miami, FL) cells were tested and authenticated [14, 45, 46]. The cells were cultured per ATCC recommendations. Primary murine mammary tumor cells (MMTV-PyMT, p18^mt^;Gata3^+/−^ and p18^mt^;Brca1^+/−^) were isolated, screened, and cultured as previously described [15, 16]. For knockdown of Gata3 in MMTV-PYMT tumor cells, cells were infected with psi-LVRU6GP-control, psi-LVRU6GP-Gata3-a, or psi-LVRU6GP-Gata3-c (GeneCopoeia, Guangzhou, China), then selected with puromycin, as previously described [16]. For knockdown of GATA3 in human tumor cells, cells were infected with pGIPZ-empty, pGIPZ-shGATA3-E9, and pGIPZ-shGATA3-B12 as previously described [16]. For stable expression of Gata3 or GATA3 in murine mammary tumor cells (p18^mt^;Gata3^+/−^ and p18^mt^;Brca1^+/−^) or human breast cancer cells (MDA-MB231), cells were infected with plvx-Flag and plvx-Flag-Gata3, or pBabe-pure-empty and pBabe-pure-GATA3, then selected with hygromycin or puromycin. For stable expression of c-Fos, p18^mt^;Gata3^+/−^ tumor cells were infected with pEZ-Lv201-empty and pEZ-Lv201-FOS, then selected with puromycin. For tumorsphere formation assay, mammary tumor cells were plated onto ultra-low attachment plates, in serum-free DMEM-F12, as previously described [36, 39]. Primary tumorspheres formed were collected and counted after 10 days of culture.

### Mice, histopathology, and immunostaining

The generation of p18^mt^ (p18^−/−^ and p18^+/−^), p18^mt^;Gata3^+/−^ (p18^−/−^;Gata3^+/−^ and p18^+/−^;Gata3^+/−^), and p18^mt^;Brca1^+/−^ (p18^−/−^;Brca1^+/−^ and p18^+/−^;Brca1^+/−^) mice has been previously described [15, 16]. MMTV-PyMT and NCG were purchased from GemPharmatech (Nanjing, China), and NSG mice were purchased from Jackson Laboratory (Maine, USA). The Institutional Animal Care and Use Committee at the University of Miami and Shenzhen University approved all animal procedures. Animals were housed in a specific pathogen-free environment. The investigators were not blinded to genotype allocation during experiments and outcome assessment. No randomization method was used as mice were segregated into groups based on genotype. Histopathology, immunohistochemistry (IHC), and immunofluorescence staining (IF) were performed as previously described [14, 20, 36]. The primary antibodies used were GATA3, FRA1, c-FOS, SNAIL, TWIST, E-cadherin (E-cad), Ki67, Fibronectin (Fn), Vimentin (Vim) (Cell Signaling). Immunocomplexes were detected using the Vectastain ABC alkaline phosphatase kit according to the manufacturer’s instructions (Vector Laboratories), or using FITC- or rhodamine-conjugated secondary antibodies (Jackson Immunoresearch).

### CRISPR-mediated Fra1 knockout, transplantation, and analysis of tumor initiation and metastasis

For CRISPR-mediated Fra1 knockout in p18^mt^;Gata3^+/−^ primary tumor cells, Fra1 Double Nickase and control Double Nickase plasmids (Santa Cruz) were transfected into p18^mt^;Gata3^+/−^ primary tumor cells, respectively, following the manufacturer’s protocol. Three days after selection with puromycin, GFP-positive cells were FACS sorted for further analysis, as previously described [39]. For mammary fat pad (MFP) transplantation, tumor cells were suspended in a 50% solution of Matrigel (BD) and then inoculated into the left and right inguinal MFPs of 4–6-week-old female NCG or NSG mice, respectively, in a pairwise manner, as previously described [15, 16]. For analysis of tumor-initiating potential, p18^mt^ and p18^mt^;Gata3^+/−^ mammary tumor cells were inoculated into the MFPs of NSG mice with subcutaneous implantation of estrogen pellets. Eight or sixteen weeks after transplantation, mice with tumor larger than 15 mm^3^ were counted. For analysis of tumor metastatic potential, 10^6^ p18^mt^;Gata3^+/−^ or p18^mt^;Brca1^+/−^ tumor cells were injected via the tail vein into NCG mice. Four weeks after tail vein injection, the lungs were surgically removed, fixed, and assessed using hematoxylin (H.E) staining. The number of metastatic nodules in the lungs was quantified as we previously described [39]. Briefly, fixed lung tissues of all five lobes were sagittally sectioned at 200-μm intervals. At least three sections for each lobe were prepared and stained with H.E. The metastatic nodules in each lobe of lung tissue were confirmed by H.E. staining, counted under a microscope, and averaged. The number of nodules in all lobes was then calculated.

### Western blot and qRT-PCR

Tissue and cell lysates were prepared as previously reported [36]. Primary antibodies used were as follows: GATA3, FRA1, c-FOS, HSP90, E-cad, Fn, Vim (Cell Signaling), β-actin, GAPDH (Biosharp). For qRT-PCR, total RNA was extracted with Trizol reagent according to the manufacturer’s protocol, and cDNA were synthesized using Hifair II 1^st^ Strand SuperMix (YEASEN, Shanghai, China). Real-time PCR was performed as previously reported [15, 16]. Primers used are listed in Table S1.

### Cell migration and invasion assay

Cell migration and invasion assays were carried out using Transwell chambers with 8-μm pore membrane (Corning) and Invasion chambers (Corning), respectively, according to the manufacture’s instruction. Briefly, 2 × 10^4^ MDA-MB-231 cells infected with pBabe-puro-empty (Empty) and pBabe-puro-GATA3 (GATA3) and serum-starved for 24 h were seeded into the upper chamber in 2% FBS-containing medium. 20% FBS-containing medium was added into the lower chamber. 24 or 48 h later, the medium and the cells remaining on the upper surface of the membrane were removed with cotton swabs. The cells on the lower surface of the membrane were fixed and stained with 0.1% crystal violet. The cells in five randomly chosen microscopic fields were counted and averaged.

### Chromatin-immunoprecipitation (CHIP) assay

ChIP assays were performed as previously described [39]. Briefly, T47D cells were treated with 1.5% formaldehyde and sonicated. Anti-GATA3 antibody (Cell Signaling) or control mouse IgG was used to precipitate chromatin associated with GATA3. Q-PCR was performed to determine the relative abundance of target DNA. Specific primers for the analysis of GATA3 binding to FOSL1 or FOS are listed in Table S1.

### Dual-luciferase reporter assay

The human FOSL1 promoter region −1138 bp to −724 bp that covers P1 primers used for ChIP analysis and contains three GATA3 binding sites with consensus sequences, GATAA, at −944, −924, and −826 was inserted into the pGL3-basic (Promega), as pGL3-FOSL1. The human FOS promoter region −2286 bp to −679 bp that covers P1, P2, and P3 primers used for ChIP analysis and contains four GATA3 binding sites with consensus sequences, GATAA, at −930 and −1062, GATAG at −1646, and TGATTA at −2236 was inserted into the pGL3-basic (Promega), as pGL3-FOS. For promoter-luciferase reporter assay, we infected MDA-MB231 cells with pLvx-Flag (Empty) and pLvx-Flag-GATA3 (GATA3) and established empty- and GATA3-expressing stable cells, which were then transfected with Renilla vector (internal control), pGL3-basic, pGL3-FOSL1, or pGL3-FOS. 48 h after transfection, cell lysates were collected and subjected to luciferase assay using the Dual-Luciferase Reporter Assay System (Promega). Two independent transfection experiments were conducted, and each luciferase assay was performed in triplicates. Normalized data was calculated as the ratio of the firefly/Renilla luciferase activities.

### Human tumor samples and meta-analysis of gene expression datasets

Formalin-fixed paraffin-embedded (FFPE) human breast cancer samples lacking patient-identifying information were obtained from the Tissue Bank Core Facility at the University of Miami and the Department of Pathology at Shenzhen University. Samples used for this study consisted of non-treated invasive breast carcinomas with known ER status, as previously reported [36, 38], as well as breast carcinoma along with paired lymph node metastasis. The GOBO breast cancer database (http://co.bmc.lu.se/gobo/gsa.pl) was analyzed to compare gene expression versus five molecular subtypes of breast cancer. Breast Cancer Gene-Expression Miner v4.8 database (bcGenExMiner v4.8; http://bcgenex.ico.unicancer.fr/BC-GEM/GEM-Accueil.php?js=1) was analyzed to compare gene expression versus ER positive and negative subtypes of breast cancer. The correlation of expression of GATA3 mRNA with FOSL1 and FOS mRNA was analyzed with TCGA [23] and bcGenExMiner v4.8 human breast cancer datasets. Prognostic values of GATA3, FOSL1, and FOS expression were assessed by displaying the distant metastasis-free survival (DMSF) using the Kaplan–Meier plotter integrative data analysis tool (www.kmplot.com).

### Statistical analysis

All data are presented as the mean ± SD for at least three repeated individual experiments for each group. Sample sizes and normalization methods are indicated in each figure legend. Statistical analyses were performed as described in each figure legend using GraphPad PRISM 6.02 software. Quantitative results were analyzed by the two-tailed Fisher exact test or two-tailed Student’s t test. *p* < 0.05 was considered statistically significant.

## Supplementary information

Table S1, Figure S1-S9

Full and uncropped western blots

Checklist

## Data Availability

All data generated or analyzed during this study are included in this published article and its supplementary information files.
