# Supplementary material for: Loss of function of GATA3 regulates FRA1 and c-FOS to activate EMT and promote mammary tumorigenesis and metastasis
Source: Cell Death Dis. 2023 Jun 23;14(6):370. doi: 10.1038/s41419-023-05888-9 (PMC10290069; doi:10.1038/s41419-023-05888-9)
Supplement: Supplementary file 1 — Table S1, Figure S1-S9 [file 41419_2023_5888_MOESM1_ESM.pdf]

Table S1
List of primer sequence

	Species	FW/RV	Sequences
**Primers for ChIP GATA3 on FOS locus**			
P1	Human	FW	CTTCAAATGTCTTCGCACGTA
		RV	GGAAGCAGAAAGTGGAGGATC
P2	Human	FW	GCTGTGTTGCTGTAAACAG
		RV	TCTTCCCTTCTCCACTCTTC
P3	Human	FW	CTTGACCTATTTTGTCTGAAG
		RV	ACGGAAAAACACACATAATAC
P4	Human	FW	CAACTCTGGTCATAATGGTG
		RV	CAACAGAGCAAGATTCTGTCTC
P5	Human	FW	TAGGCTACCTTGTGTGAGCT
		RV	AGAGGAGGTGACTGCAATACA
P6	Human	FW	TCTATCGGATGAGGAAACACT
		RV	GATTTCATGCTCTGGACAAGT
P7	Human	FW	ATCATGAACCACTGGGGTGT
		RV	AGGTCTGGCAATAAATATCTC
**Primers for ChIP GATA3 on FOSL1 locus**			
P1	Human	FW	CAGCTACTCACTTAGCGACT
		RV	GATTGGGTCTCGCCGTATTG
P2	Human	FW	CAGCCTATGTGTCAAATTCT
		RV	GAATGTGGTTAGGCTGCTCA
P3	Human	FW	TAGGCATCTAGGCATCAGAGT
		RV	CTTGGTGGCTCTGTAAGACT
**Primers for qRT-PCR**			
Gata3	Mouse	FW	GCTGACGGAAGAGGTGGACGTACT
		RV	TGGTGGGTCGGAGGATACCT
Fos	Mouse	FW	CGAAGGGAACGGAATAAGATG
		RV	GCTGCCAAAATAAACTCCAG
Fosl1	Mouse	FW	GAGACGCGAGCGGAACAAG
		RV	CTTCCAGCACCAGCTCAAGG
Cdh1	Mouse	FW	AACAACTGCATGAAGGCGGGAATC
		RV	CCTGTGCAGCTGGCTCAAATCAAA
Vim	Mouse	FW	CCAACCTTTTCTTCCCTGAA
		RV	TGAGTGGGTGTCAACCAGAG
Erα	Mouse	FW	GCAAGTGTTACGAAGTGGGCATG
		RV	GCAGCCCTCATGTCTCCTGAAG

Snail	Mouse	FW	TTGTGTCTGCACGACCTGTGGAAA
		RV	TCTTCACATCCGAGTGGGTTTGGA
Slug	Mouse	FW	CACATTCGAACCCACACATTGCCT
		RV	TGTGCCCTCAGGTTTGATCTGTCT
Twist	Mouse	FW	CGGGTCATGGCTAACGTG
		RV	CAGCTTGCCATCTTGGAGTC
Gapdh	Mouse	FW	GGTGAAGGTCGGTGTGAACG
		RV	TGTAGACCATGTAGTTGAGG
GATA3	Human	FW	CACAACCACACTCTGGAGGAG
		RV	GTCCTCCAGTGAGTCATGCAC
FOS	Human	FW	GAGGGGCAAGGTGGAACAGT
		RV	CTTGCAGGCAGGTCGGTGAG
FOSL1	Human	FW	GGGCCTGTGCTTGAACCTGA
		RV	TCTCCGCTGCTGCTGCTACTC
CDH1	Human	FW	CCAGGAGCCAGACACATTTATGG
		RV	CTGTGTACGTGCTGTTCTTCACG
VIM	Human	FW	GAGAACTTTGCCGTTGAAGC
		RV	GCTTCCTGTAGGTGGCAATC
GAPDH	Human	FW	AGGTGAAGGTCGGAGTCAAC
		RV	AGTTGAGGTCAATGAAGGGG

Fig. S1

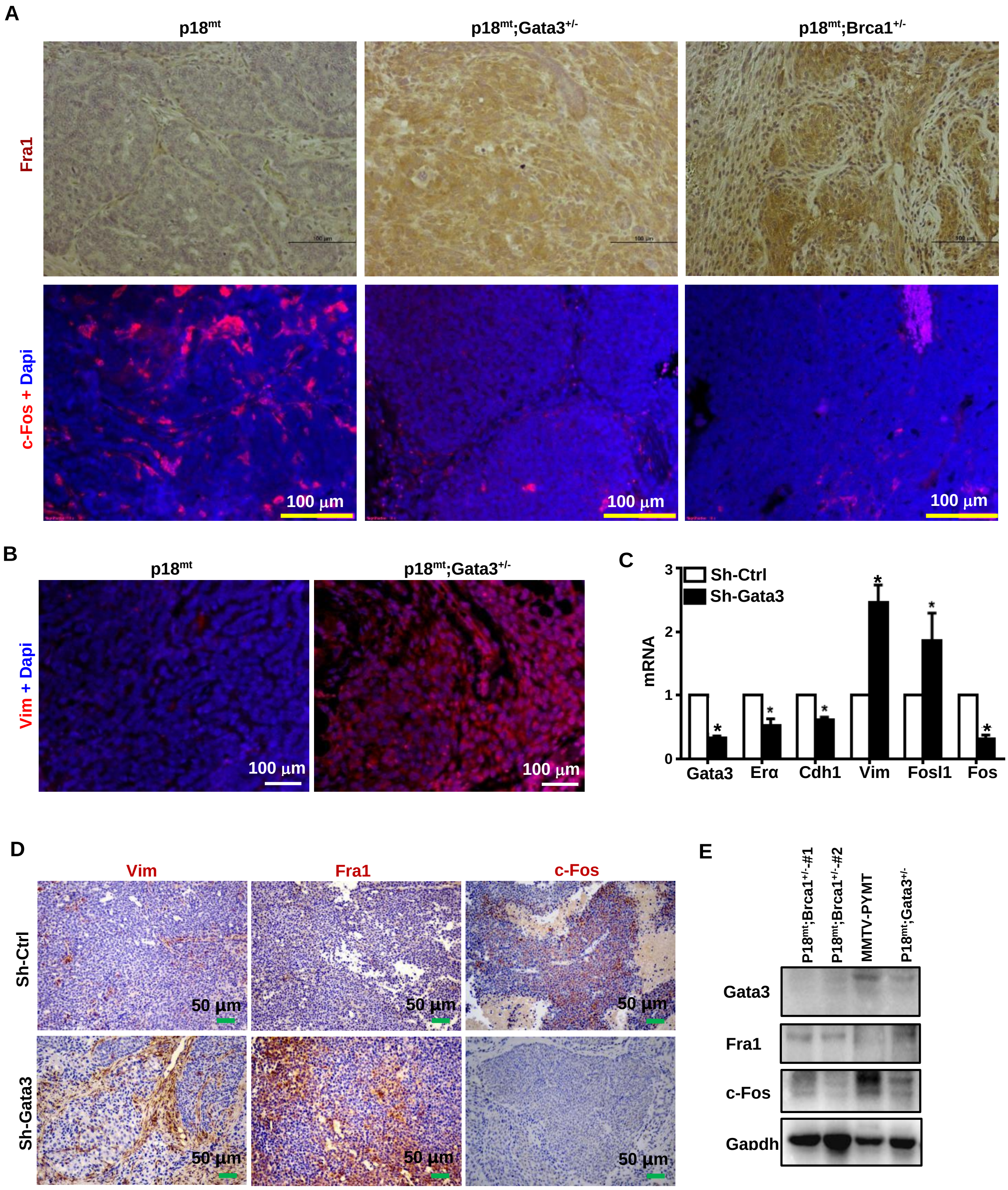
A
p18mt
p18mt;Gata3+/-
p18mt;Brca1+/-
Fra1
c-Fos + Dapi
100 μm
100 μm
100 μm
B
p18mt
p18mt;Gata3+/-
Vim + Dapi
100 μm
100 μm
C
Sh-Ctrl
Sh-Gata3
mRNA
3
2
1
0
Gata3
Erα
Cdh1
Vim
Fosl1
Fos
D
Vim
Fra1
c-Fos
Sh-Ctrl
Sh-Gata3
50 μm
E
P18mt;Brca1+/--#1
P18mt;Brca1+/--#2
MMTV-PYMT
P18mt;Gata3+/-
Gata3
Fra1
c-Fos
Gapdh

Fig. S1. Analysis of Gata3-deficient and Brca1-deficient mammary tumors.

(A, B) Representative IHC and IF analysis of primary mammary tumors with antibodies against Fra1, c-Fos (A), and VIM (B). (C, D) MMTV-PyMT mammary tumor cells were infected with psi-LVRU6GP-empty (sh-Ctrl) or psi-LVRU6GP-Gata3 (sh-Gata3), and then analyzed by qRT-PCR (C) or transplanted into MFPs of NCG mice. Tumors generated by sh-Ctrl and sh-Gata3 cells were analyzed by IHC (D). Data in (C) represent the mean ± SD from duplicates of two independent experiments. The asterisk (*) denotes a statistical significance from sh-Ctrl and sh-Gata3 samples determined by a two-tailed, paired T test. (E) Analysis of representative mammary tumors with the indicated genotype by western blot.

Fig. S2

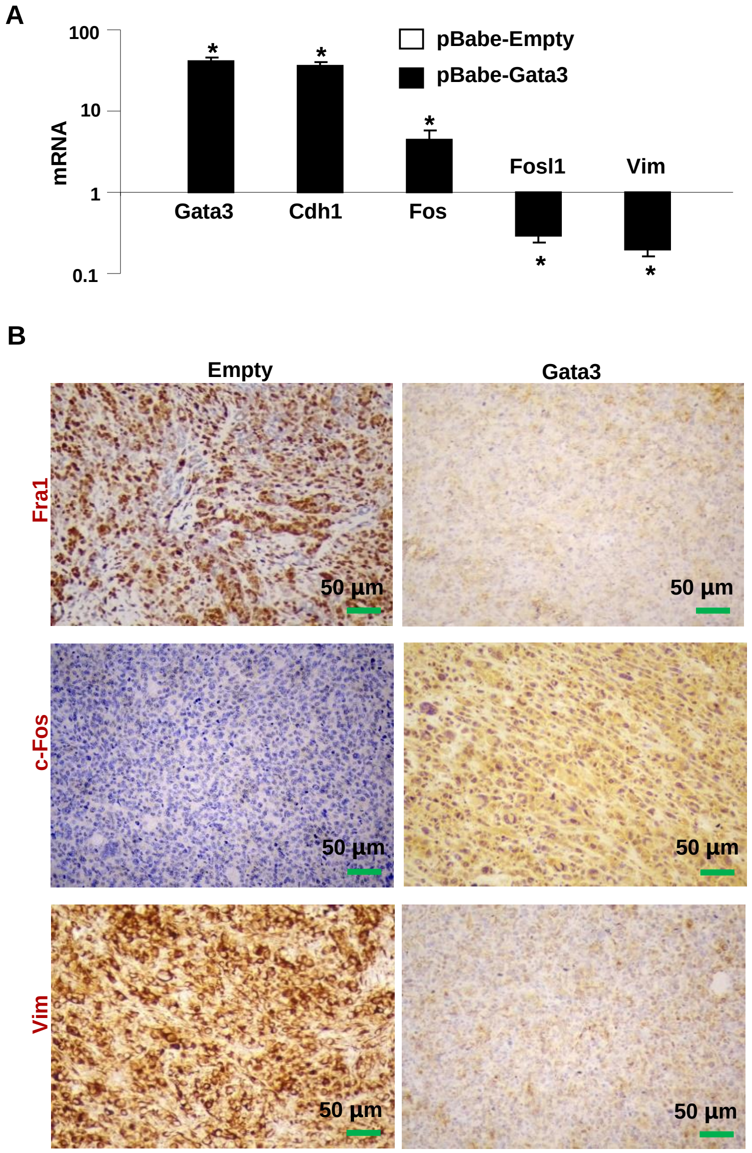

Fig. S2. Reconstitution of Gata3 in Brca1-deficient tumor cells stimulates c-Fos and suppresses Fra1 expression in inhibition of EMT and mammary tumorigenesis

p18^{mt};Brca1$^{+/-}$ tumor cells were infected with pBabe-Empty (Empty) and pBabe-Gata3 (Gata3), and then analyzed by qRT-PCR (A). Data represent the mean ± SD from duplicates of two independent experiments. The asterisk (*) denotes a statistical significance from Empty and Gata3 samples determined by a two-tailed, paired T test. (B) p18^{mt-};Brca1$^{+/-}$ tumor cells infected with pBabe-Empty (Empty) or pBabe-GATA3 (Gata3) were transplanted into the left and right inguinal MFPs of five female NCG mice, respectively, in a pairwise manner. Tumors regenerated were analyzed by IHC.

Fig. S3

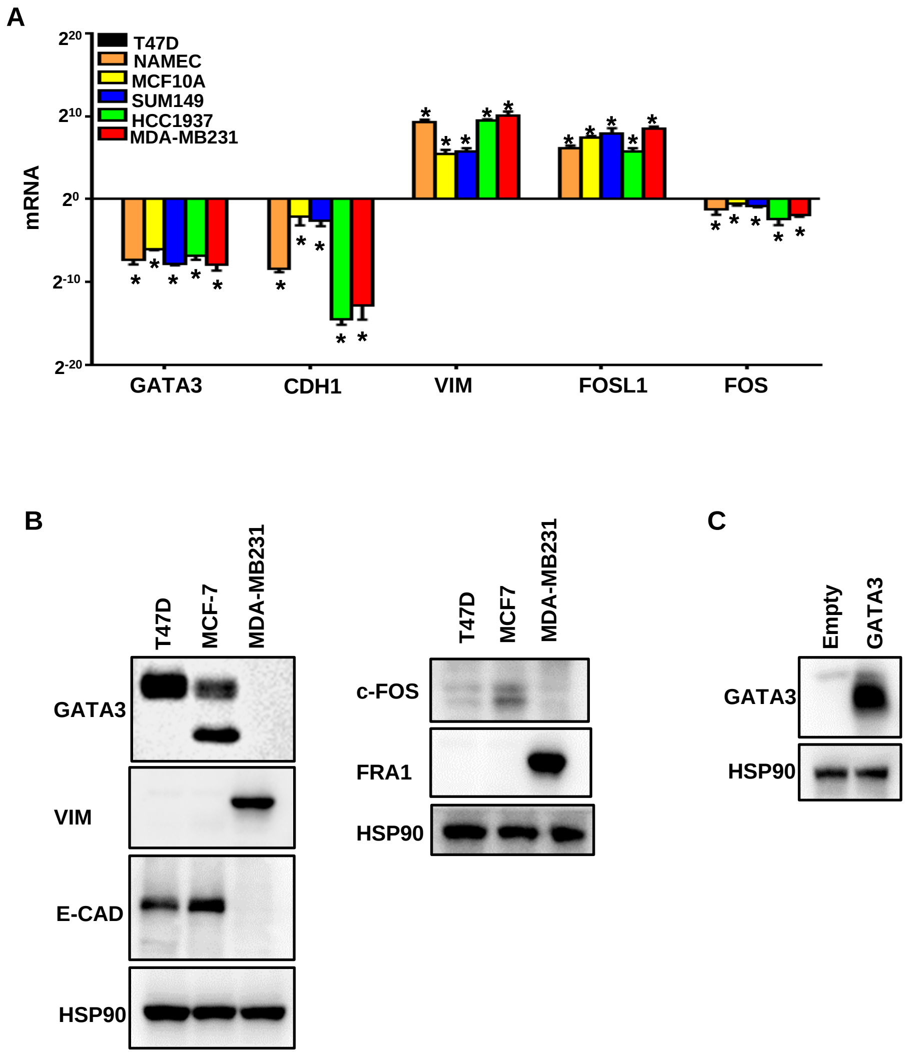

Fig. S3. Analysis of the expression of FRA1 and FOS in human breast cell lines.

(A, B) Analysis of human breast cell lines by qRT-PCR (A) and western blot (B). NAMEC, a naturally arising mesenchymal cell line from HMLE cells that had spontaneously undergone an EMT. MCF10A, a basal epithelial cell line. Data in (A) represent the mean ± SD from duplicates of two independent experiments. The asterisk (*) denotes a statistical significance from T47D and individual cell lines determined by a two-tailed, unpaired T test. (C) MDA-MB231 were infected with pBabe-puro-empty (Empty) and pBabe-puro-GATA3 (GATA3), selected with puromycin, and analyzed by Western blot.

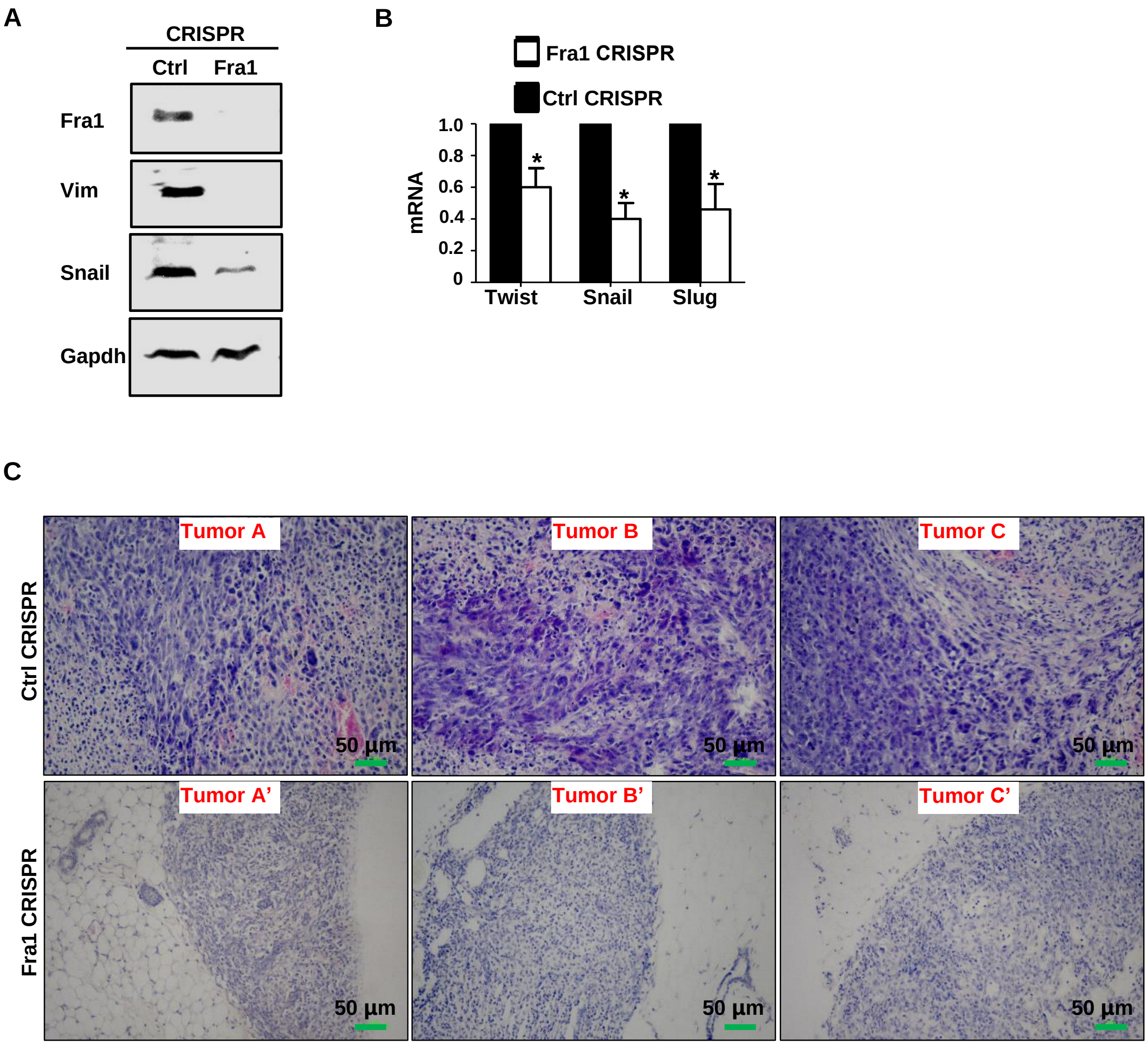

Fig. S4. Fra1 knockout inhibits EMT in Gata3-deficient mammary tumor cells and suppresses Gata3-deficient tumorigenesis.

$p18^{mt}$;$Gata3^{+/-}$ tumor cells were transfected with Fra1 (Fra1 CRISPR) and Control (Ctrl CRISPR) Double Nickase plasmids, selected with puromycin for 3 days, and then analyzed by western blot (A) and qRT-PCR (B). The asterisks (*) denote a statistical significance from Fra1 CRISPR and Ctrl CRISPR cells determined by a two-tailed, paired T test. (C) 1 x 104 Fra1- and Ctrl-knockout p18mt;Gata3+/- mammary tumor cells were inoculated into the left and right inguinal MFPs of NSG mice, respectively, in a pairwise manner. Four months after transplantation, mice were dissected and the tumors regenerated were analyzed by H.E staining.

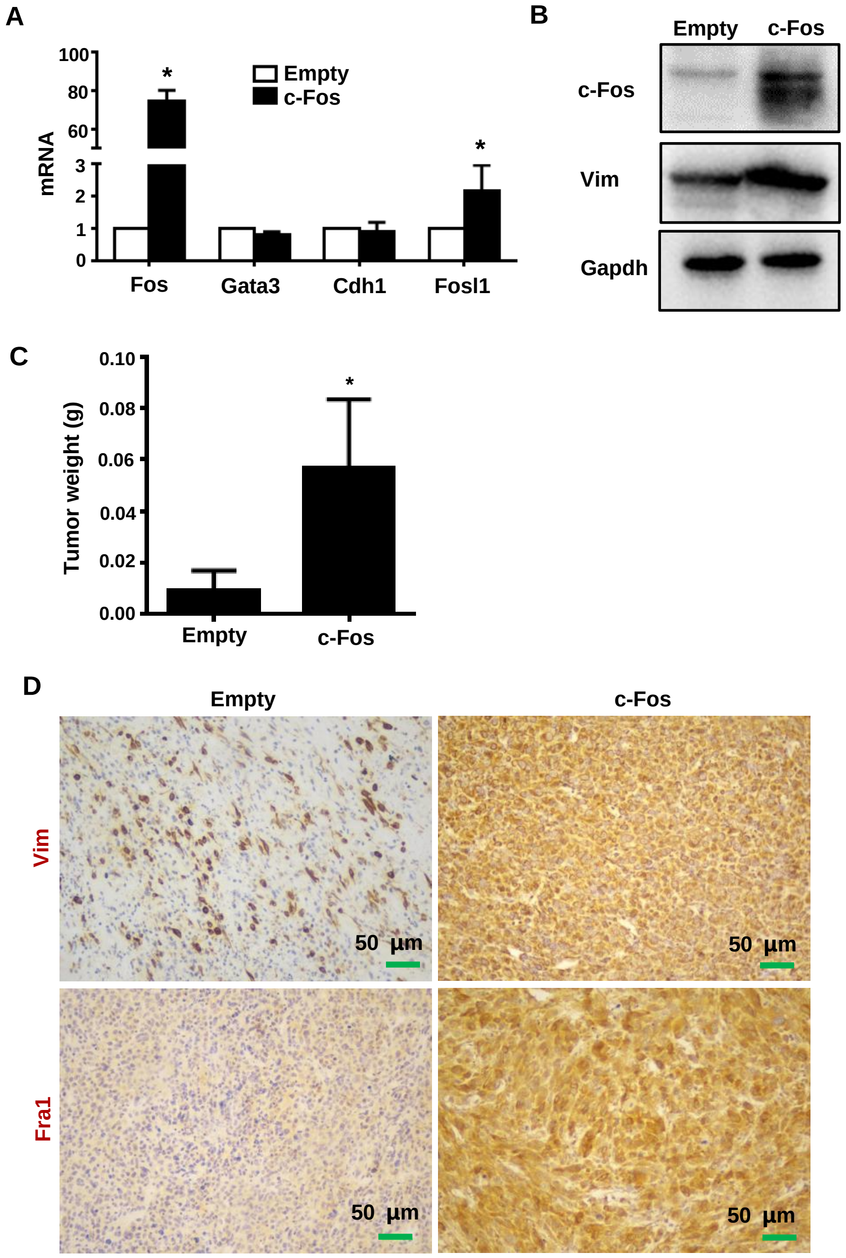

Fig. S5. Reconstitution of c-Fos in Gata3-deficient tumor cells stimulates the expression of Fra1 and promotes tumorigenesis. (A, B) Another p18^{mt};Gata3$^{+/-}$ mammary tumor cell line (p18^{mt};Gata3$^{+/-}$-2) derived from an additional p18^{mt};Gata3$^{+/-}$ mouse were infected with pEZ-Lv201 (empty) and pEZ-Lv201-c-Fos (c-Fos), selected with puromycin, and analyzed by qRT-PCR (A) and western blot (B). The asterisk (*) denotes a statistical significance from empty and c-Fos samples determined by a two-tailed, paired T test. (C, D) p18^{mt};Gata3$^{+/-}$-2 tumor cells infected with pEZ-Lv201 (empty) and pEZ-Lv201-c-Fos (c-Fos) were transplant into the left and right inguinal MFPs of female NCG mice, respectively, in a pairwise manner. Weight of the tumors generated were determined (C), and representative tumors were analyzed by IHC (D). Data in (C) represent the mean ± SD of four tumors in each group. The asterisks (*) denote a statistical significance from c-Fos and empty tumors determined by a two-tailed, paired T test.

A

#	ER	GATA3	FRA1	c-FOS
1	+	++++	-	++
2	+	+++	-	++
3	+	+++	-	+
4	+	++	-	+
5	+	++	+/-	+
6	+	++	+	+/-
7	+	++	+/-	-
8	+	+	+/-	-
9	-	+/-	+	-
10	-	+/-	+	+/-
11	-	+/-	+/-	+
12	-	-	+/-	+/-
13	-	-	+	-
14	-	-	+	+/-
15	-	-	++	-
16	-	-	+++	-
17	-	-	+++	-
18	-	-	+++	-

B

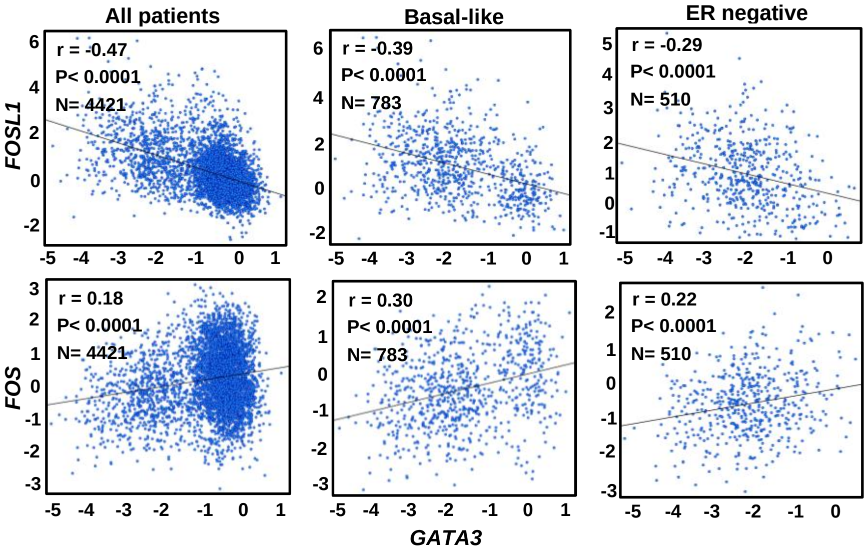

C

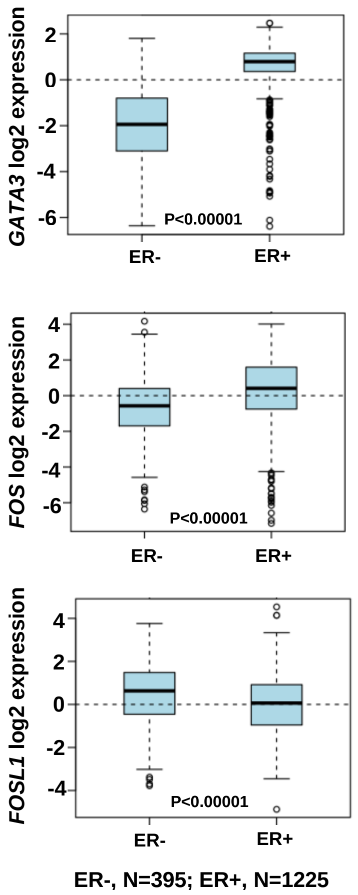

D

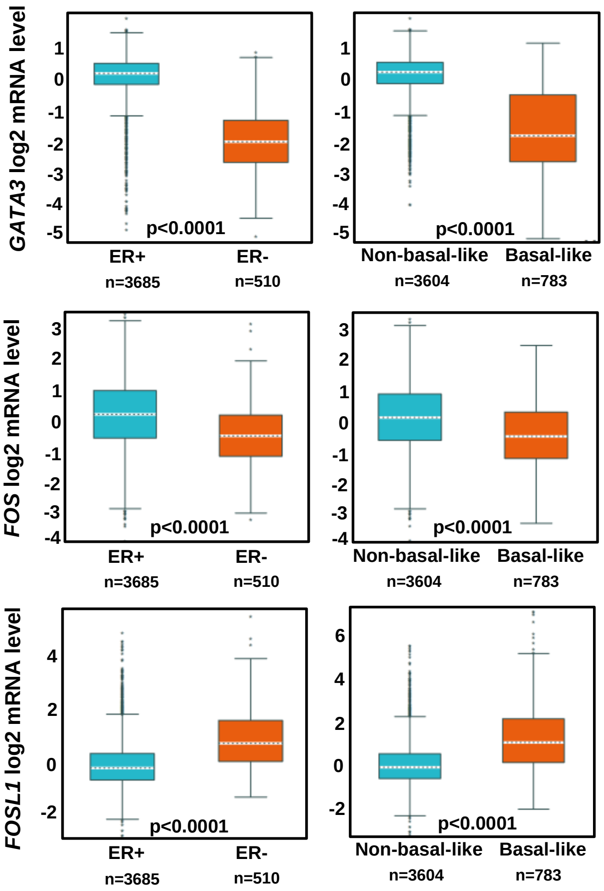

Fig. S6. Correlation analysis of GATA3 with FRA1 and c-FOS in human breast cancers. (A) Immunostaining results for individual human breast cancer tumors shown in 7D and 7E. +/-, <2%; +, 2-10%; ++, 10-40%; +++, 40-70%; ++++, >70%. (B) Correlation analysis of *GATA3* and *FOSL1* or *FOS* in bcGenExMiner v4.8 breast cancer database. (C, D) Analysis of gene expression of *GATA3, FOSL1 and FOS* in GOBO breast cancer database and bcGenExMiner v4.8 database according to subtype.

Fig. S7

A

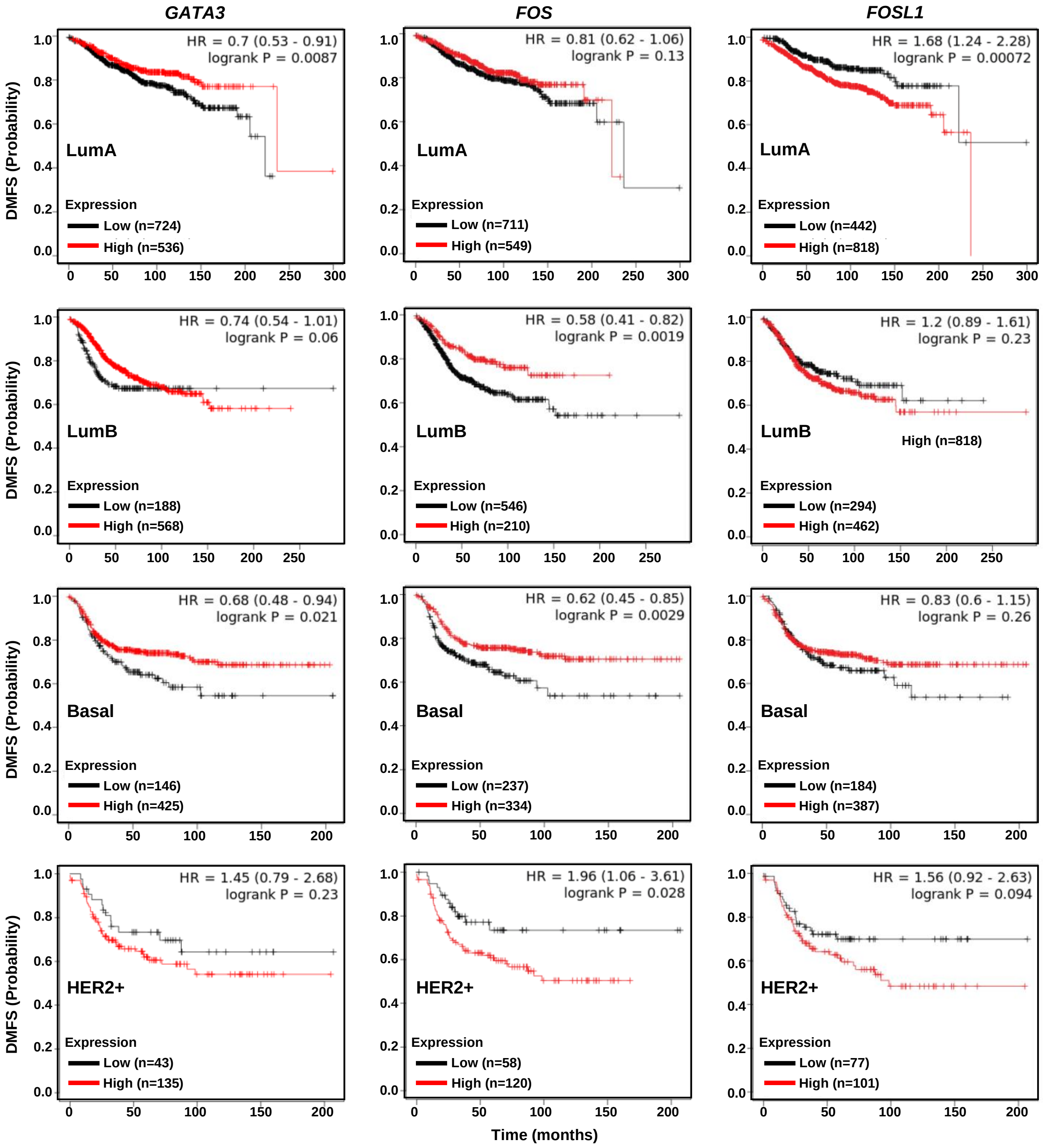
GATA3
FOS
FOSL1
HR = 0.7 (0.53 - 0.91)
logrank P = 0.0087
LumA
Expression
Low (n=724)
High (n=536)
HR = 0.81 (0.62 - 1.06)
logrank P = 0.13
LumA
Low (n=711)
High (n=549)
HR = 1.68 (1.24 - 2.28)
logrank P = 0.00072
LumA
Low (n=442)
High (n=818)
HR = 0.74 (0.54 - 1.01)
logrank P = 0.06
LumB
Low (n=188)
High (n=568)
HR = 0.58 (0.41 - 0.82)
logrank P = 0.0019
LumB
Low (n=546)
High (n=210)
HR = 1.2 (0.89 - 1.61)
logrank P = 0.23
LumB
High (n=818)
Low (n=294)
High (n=462)
HR = 0.68 (0.48 - 0.94)
logrank P = 0.021
Basal
Low (n=146)
High (n=425)
HR = 0.62 (0.45 - 0.85)
logrank P = 0.0029
Basal
Low (n=237)
High (n=334)
HR = 0.83 (0.6 - 1.15)
logrank P = 0.26
Basal
Low (n=184)
High (n=387)
HR = 1.45 (0.79 - 2.68)
logrank P = 0.23
HER2+
Low (n=43)
High (n=135)
HR = 1.96 (1.06 - 3.61)
logrank P = 0.028
HER2+
Low (n=58)
High (n=120)
HR = 1.56 (0.92 - 2.63)
logrank P = 0.094
HER2+
Low (n=77)
High (n=101)
DMFS (Probability)
Time (months)

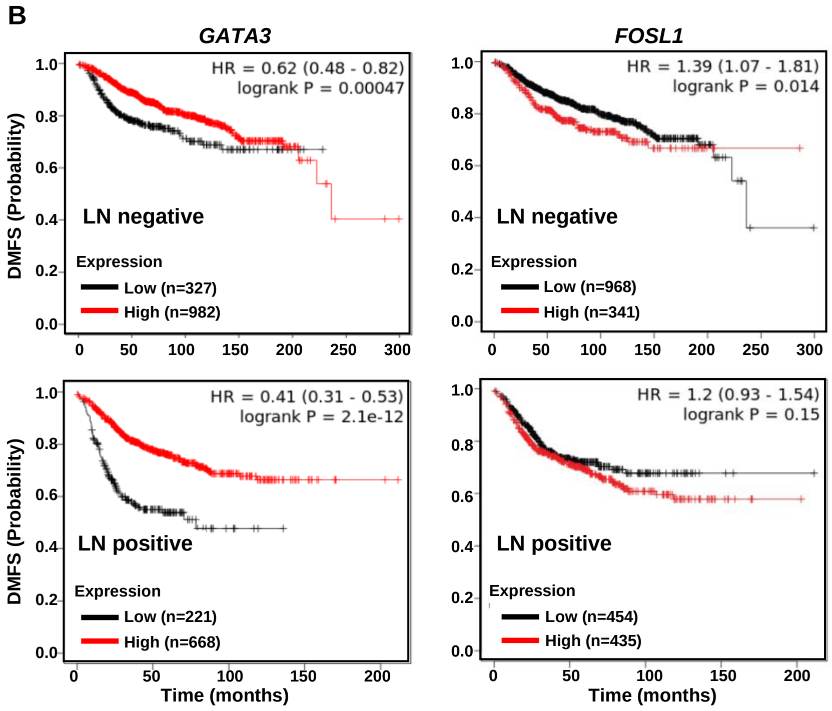

Fig. S7. The effects of GATA3, FOS, and FOSL1 expression on breast cancer patient **survival.** Kaplan–Meier plots of the distant metastasis-free survival (DMFS) were generated using the Kaplan–Meier Plotter [Breast cancer mRNA analysis tool (https://kmplot.com/analysis/)]. (A) Subtypes (Luminal A, LumA; Luminal B, Lum B; Basal-like, Basal; and HER2 positive, HER+) were selected. (B) Lymph node (LN) status was selected.

Fig. S8

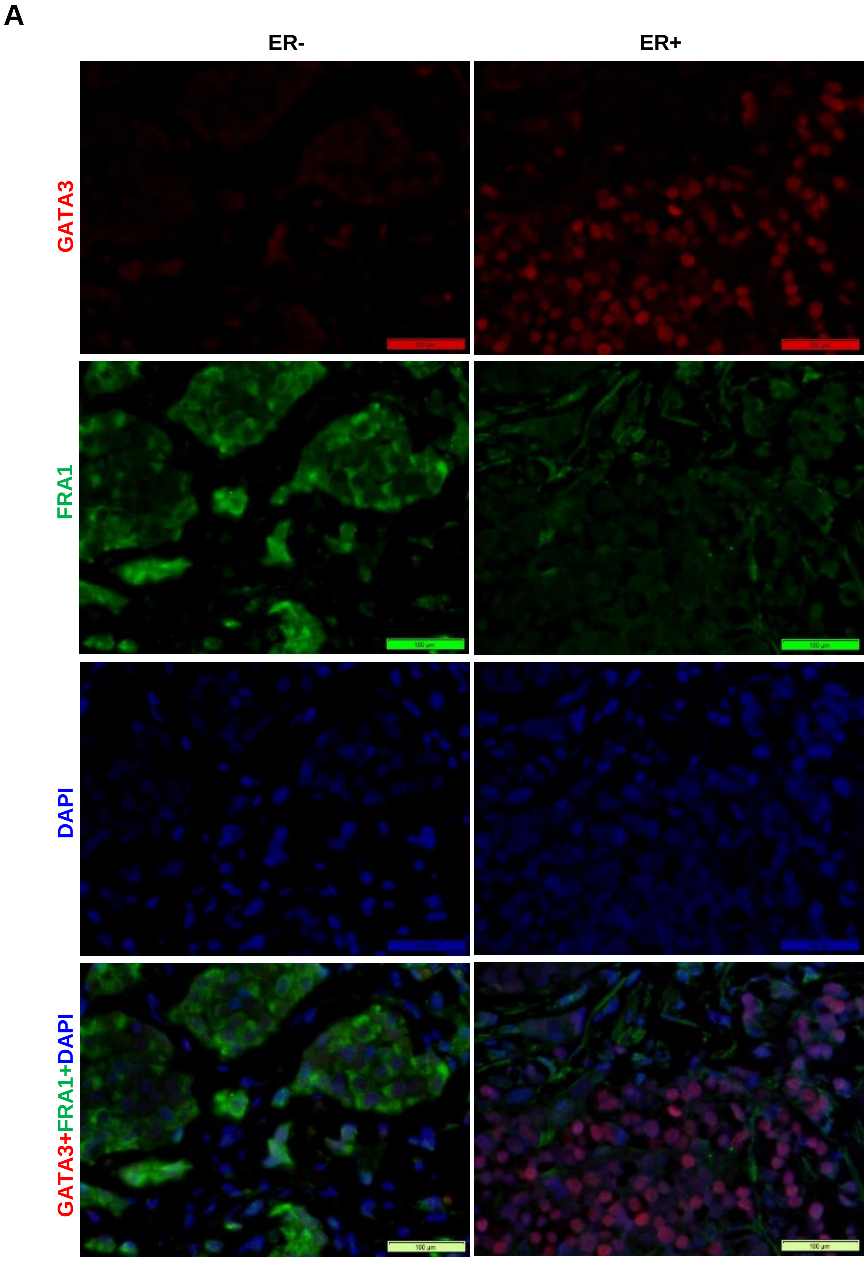

B

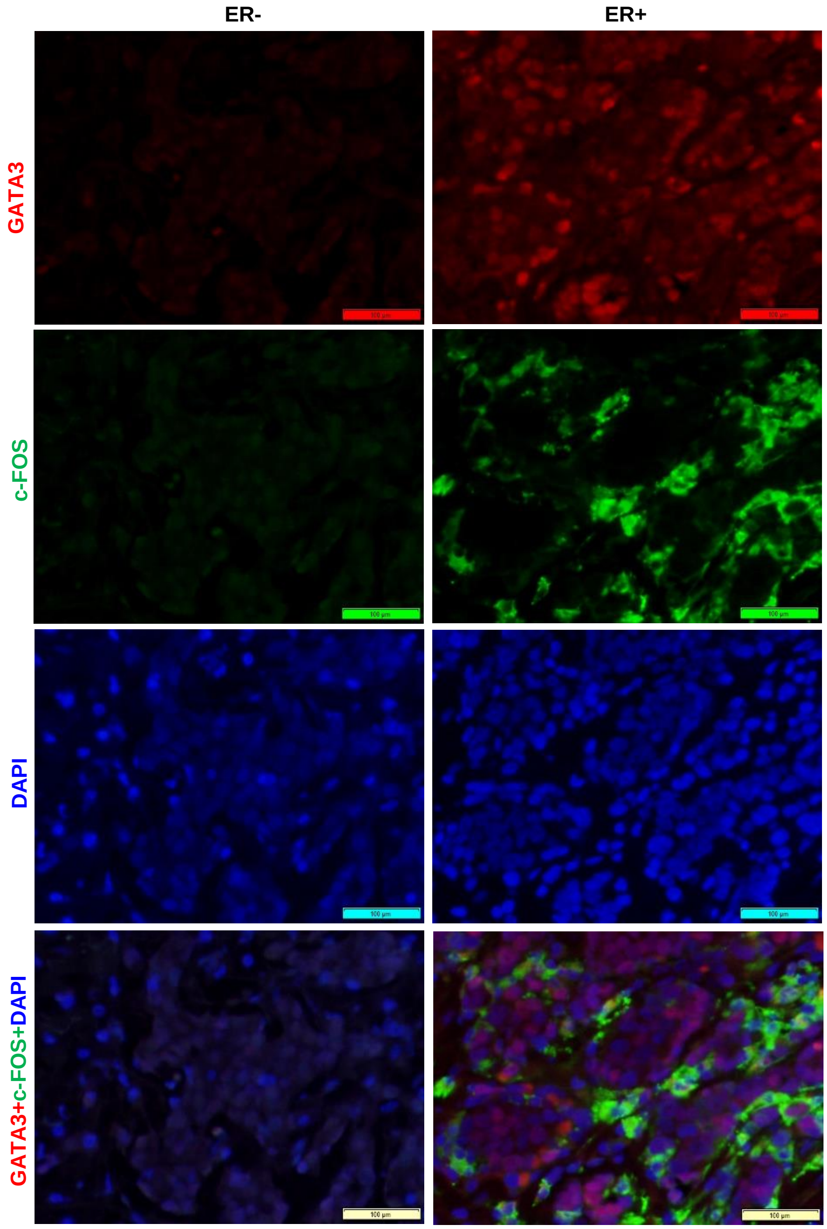

Fig. S8. IF analysis of human breast cancer samples. Representative ER-positive and ER-negative human breast cancer samples were analyzed by immunostaining with antibodies against GATA3 and FRA1 (A) or GATA3 and c-FOS (B).

Fig. S9

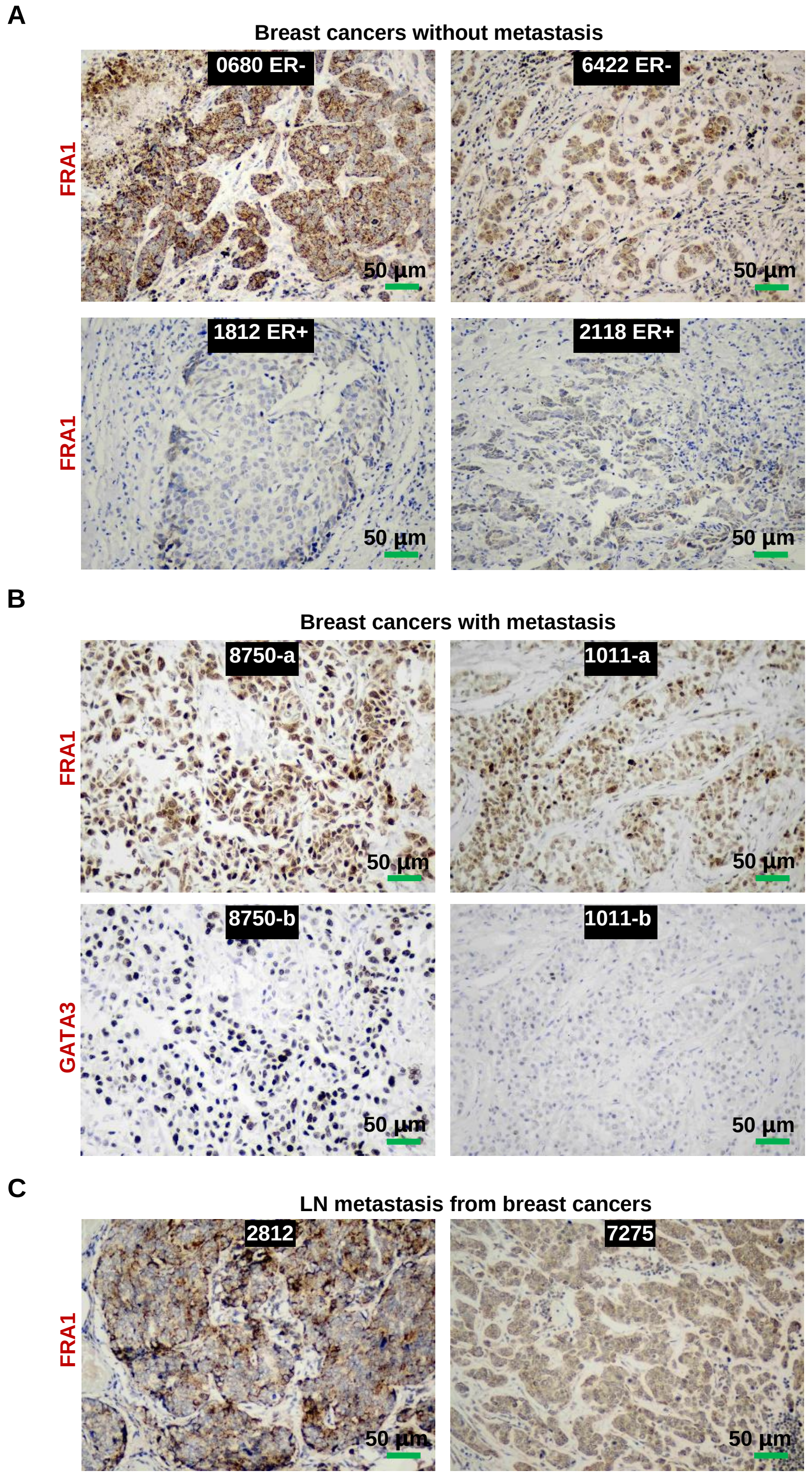

Fig. S9. IHC analysis of human breast cancers with or without lymph node metastasis. Representative IHC analysis of human breast cancers without metastasis (A), breast cancers with metastasis (B), and lymph node (LN) metastasis from breast cancers (C). Note, 8750-a and 8750-b, as well as 1011-a and 1011-b, are serial sections from the same tumor.
