# Supplementary figures and images for: Loss of function of GATA3 regulates FRA1 and c-FOS to activate EMT and promote mammary tumorigenesis and metastasis
Source: Cell Death Dis. 2023 Jun 23;14(6):370. doi: 10.1038/s41419-023-05888-9 (PMC10290069; doi:10.1038/s41419-023-05888-9)

Raw data for the blots included in each figure and supplementary data.

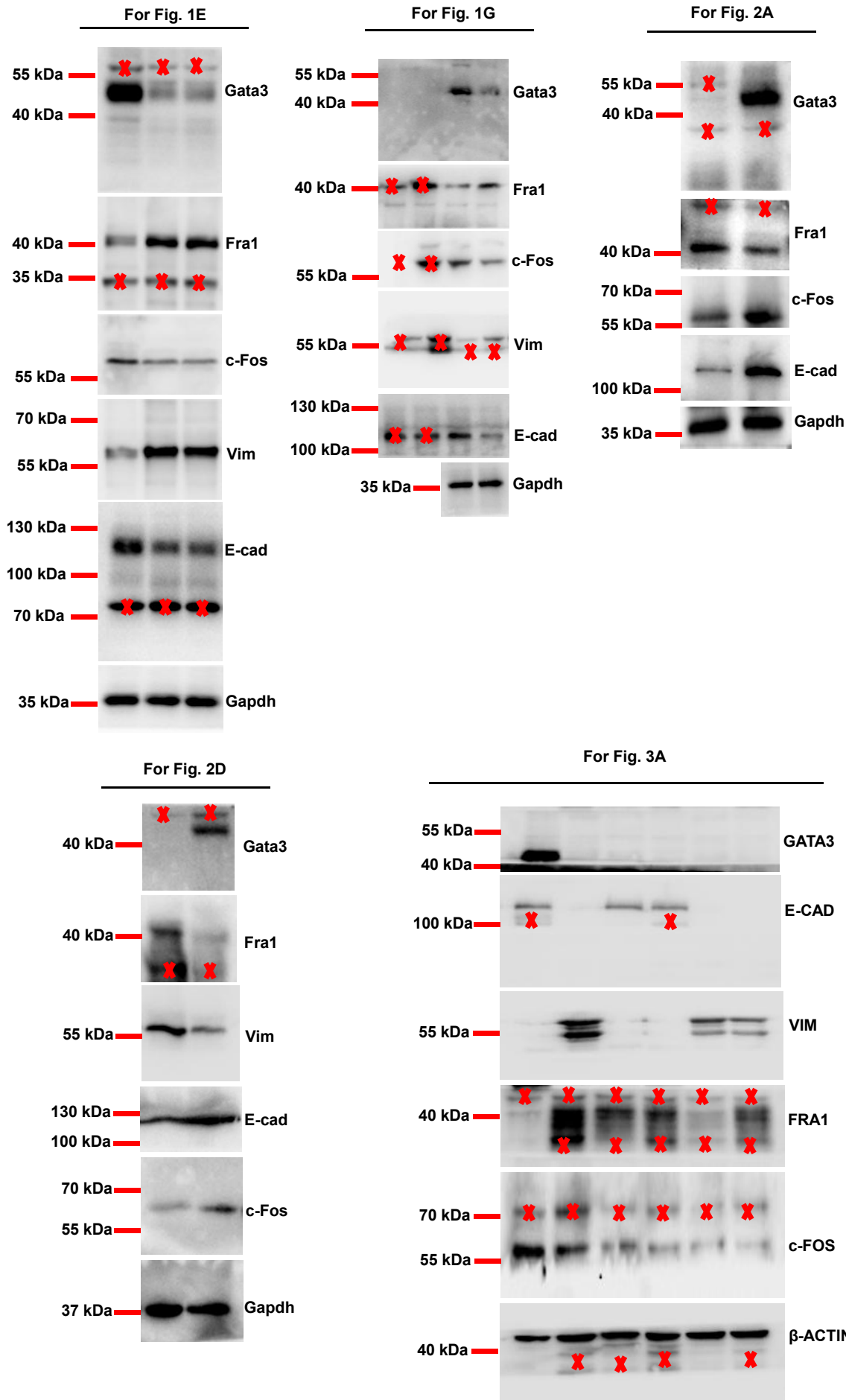

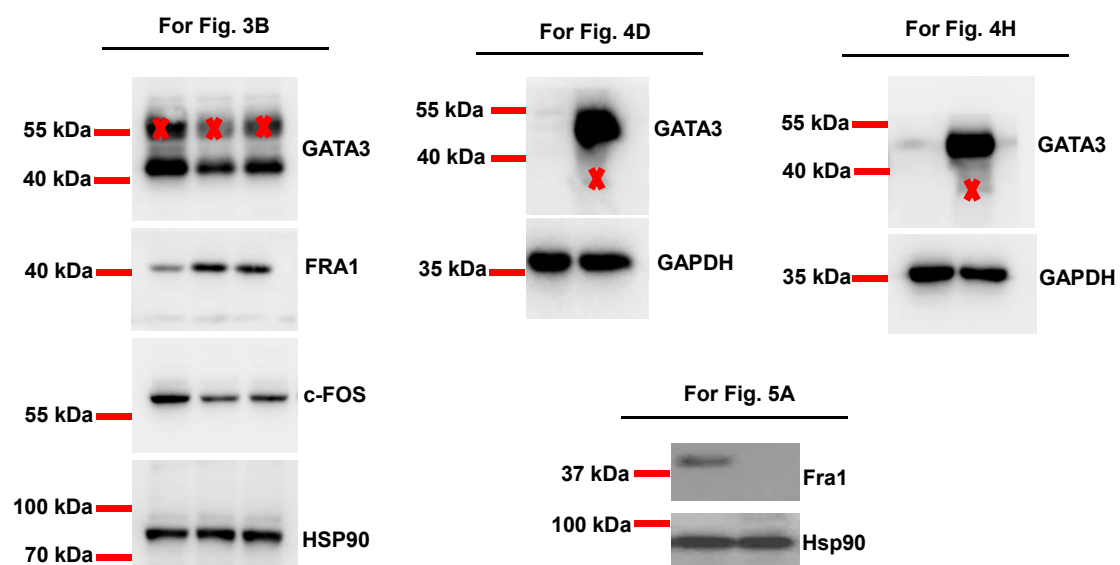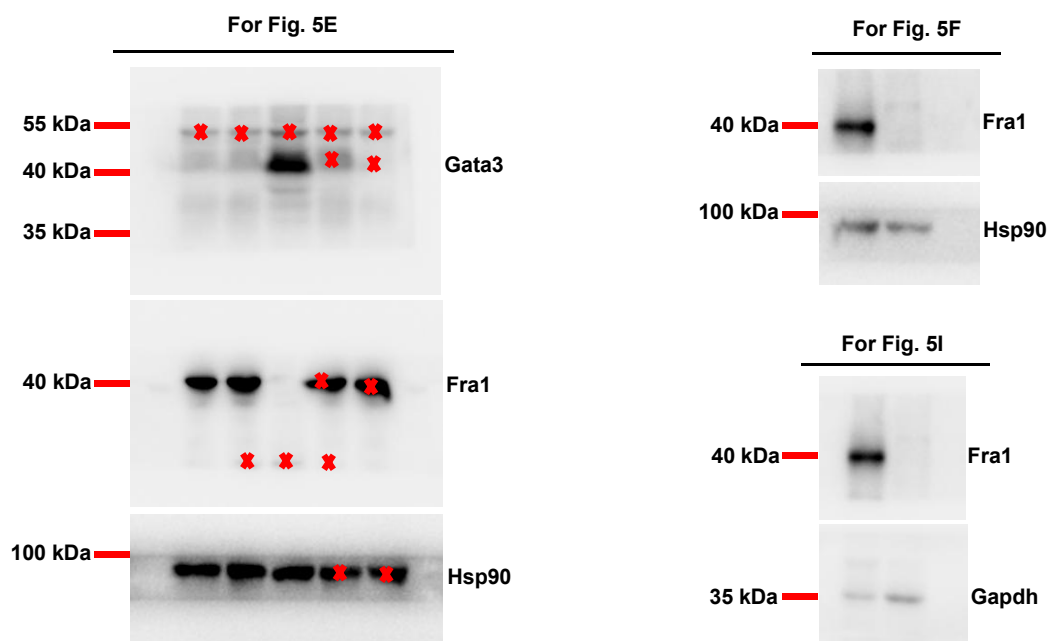

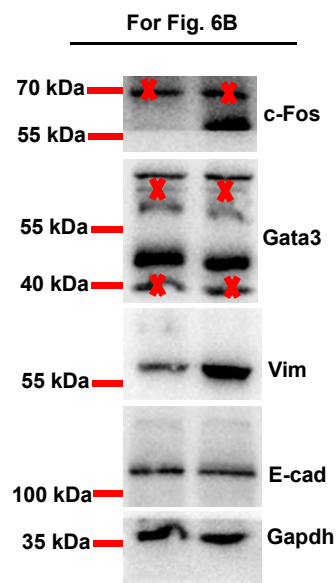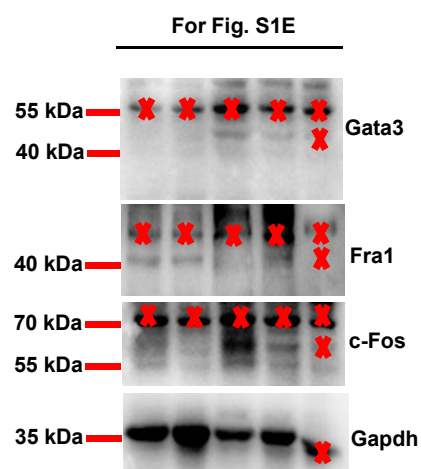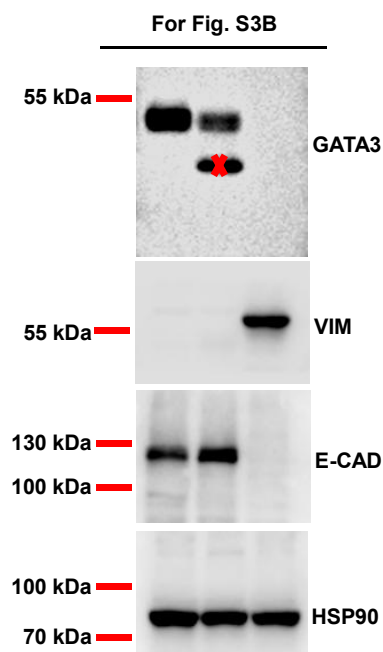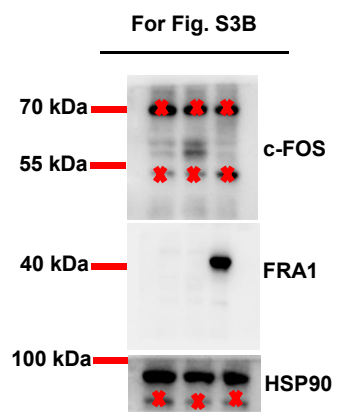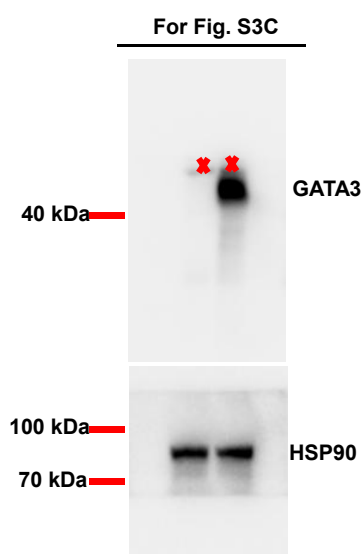

For Fig. S4A

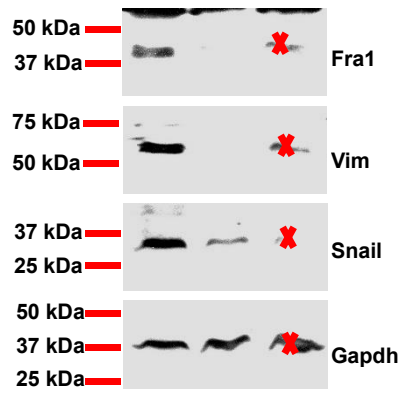

For Fig. S5B

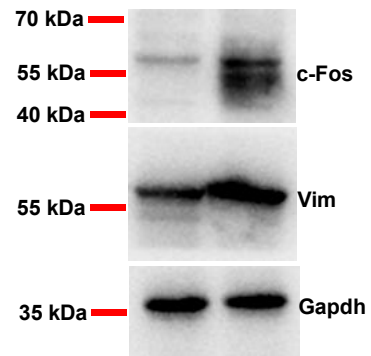

Supplement: Supplementary file 2 — Full and uncropped western blots [file 41419_2023_5888_MOESM2_ESM.pdf]
